# Multifaceted Immunomodulatory Nanocomplexes Target Neutrophilic‐ROS Inflammation in Acute Lung Injury

**DOI:** 10.1002/advs.202411823

**Published:** 2024-12-31

**Authors:** Fan Su, Chong Zhang, Qianyun Zhang, Yi Shen, Saiqi Li, Jianlin Shi, Ya‐Xuan Zhu, Han Lin, Bin He

**Affiliations:** ^1^ Department of Critical Care Medicine and Emergency, Shanghai Chest Hospital Shanghai Jiao Tong University Shanghai 200030 P. R. China; ^2^ Shanghai Tenth People's Hospital, Shanghai Frontiers Science Center of Nanocatalytic Medicine, School of Medicine Tongji University Shanghai 200072 P. R. China; ^3^ State Key Laboratory of High Performance Ceramics and Superfine Microstructure, Shanghai Institute of Ceramics Chinese Academy of Sciences; Research Unit of Nanocatalytic Medicine in Specific Therapy for Serious Disease Chinese Academy of Medical Sciences (2021RU012) Shanghai 200050 P. R. China

**Keywords:** acute lung injury, hydrogen therapy, neutrophils, reactive oxygen species

## Abstract

The sepsis‐induced acute lung injury (ALI) still represents one of the leading causes of death in critically ill patients, underscoring the need for novel therapies. Excessive activation of immune cells and damage of reactive oxygen species (ROS) are the main factors that exacerbate lung injury. Here, the multifaceted immunomodulatory nanocomplexes targeting the proinflammatory neutrophilic activation and ROS damage are established. The S100A8/9 inhibitor, ABR2575, is loaded in the nanocomplexes, which effectively blocks the neutrophils‐S100A8/A9‐ toll‐like receptors (TLRS)‐Inflammasome signaling in ALI. Synergically, the SiH nanosheets are encapsulated together with ABR2575 into the core of poly(lactic‐*co*‐glycolic acid) (PLGA) nanosponges, to achieve sustainable hydrogen release for the alleviation of ROS‐induced lung tissue injury, and also promote the M2 polarization of macrophages. This novel combination strategy is proven to significantly suppress the infiltration of neutrophils and pro‐inflammatory macrophages into the lungs, decrease the activation of neutrophils and pro‐inflammatory monocytes in the blood, facilitate the anti‐inflammatory polarization of macrophages and monocytes, and reduce the expression of pro‐inflammatory cytokines in both the lung and blood circulation, all of which alleviate the lung injuries in preclinical murine ALI models. The current investigations offer a novel nanomedicine for the treatment of ALI with great potential in clinical invention.

## Graphical abstract

This study develops immunomodulatory nanocomplexes for effective treatment of acute lung injury (ALI). By disrupting the ROS‐neutrophilic inflammation, this approach effectively alleviates the levels of oxidative stress, the infiltrations of pro‐inflammatory cells into the lung and the activations of immune cells in circulation, thereby remarkably reducing the inflammatory damage of ALI mice.

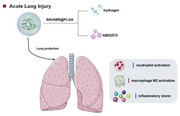

## 1 Introduction

Acute lung injury (ALI) represents one of the leading complications of patients with sepsis, and its further deterioration of hypoxia and inflammatory response leads to increased morbidity and mortality in critically ill patients.^[^ ^1^ ^]^ To date, there are no specific treatments available, emphasizing the need for new therapies. The activation of neutrophils has been considered as the hallmark of ALI, and the degree of activation in both the circulation and broncho‐alveolar lavage fluid (BALF) correlates with the severity and outcomes of ALI.^[^ ^2^ ^]^ Therefore, series investigations targeting the neutrophils activations have been performed. In particular, the alarmin protein S100A8/A9, which is mainly secreted by neutrophils and promotes the neutrophil activation, has attracted increasing attention. Inhibition of S100A8/A9 has been demonstrated to be an effective approach for the treatment of infection‐induced ALI.^[^ ^3^ ^]^ More importantly, lines of studies indicated the S100A8/A9 to be an emerging player in sepsis‐induced organ injury.^[^ ^4^ ^]^ Logically, the above findings prompt us to explore the effect of S100A8/A9 inhibition on sepsis‐induced ALI. The massive production of reactive oxygen spieces (ROS) plays another pivotal role in ALI pathological process.^[^ ^5^ ^]^ In addition to causing the direct oxidative injuries, the ROS can also increase the infiltration and pro‐inflammatory activation of macrophages and the production of pro‐inflammatory cytokines, further aggravating the inflammatory process and the injuries to the lung microenvironment.^[^ ^6^ ^]^ Therefore, the clearance of ROS to inhibit the oxidative injuries is crucial for the treatment of ALI.

Molecular hydrogen has attracted much attention since its antioxidant effect was discovered by selectively reducing cytotoxic oxygen radicals.^[^ ^7^ ^]^ Subsequent studies have proven the therapeutic effect of molecular hydrogen in various inflammatory diseases via its anti‐oxidative, anti‐inflammatory, anti‐apoptotic and various other biological effects,^[^ ^8^ ^]^ including the ALI.^[^ ^9^ ^]^ However, the poor solubility of hydrogen leads to major difficulties in storage and clinical application. Furthermore, hydrogen exhibits a concentration‐dependent effect, and may cause unexpected toxicity without controllable release. In recent years, nanomaterials capable for hydrogen delivery or generation are developed to achieve controllable and sustainable hydrogen supply in vivo, which successfully prolonged the effective time and biosafety of hydrogen therapy.^[^ ^10^ ^]^ However, these strategies usually depend on external stimuli to trigger hydrogen release,^[^ ^11^ ^]^ which limited their application scenario. Although several nanomaterials capable of generating hydrogen in biological environment without external stimuli have been developed,^[^ ^12^ ^]^ how to achieve controllable and sustainable hydrogen release still faces intractable problems.

**Figure 1:**
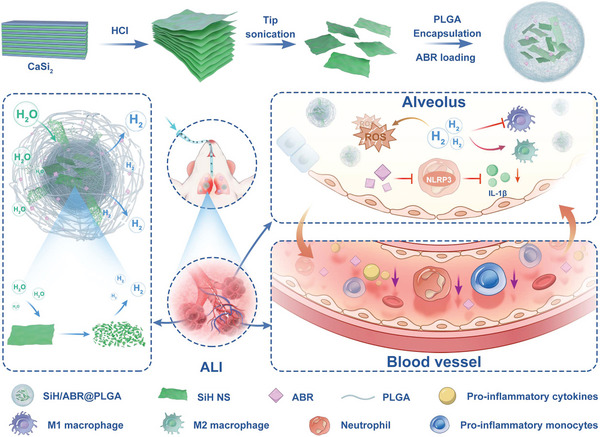
Schematic illustrating the fabrication procedure of SiH/ABR@PLGA nanosponge and its mechanism in ALI treatment.

Herein, we established a SiH‐based nanosponge (denoted as SiH/ABR@PLGA) for in situ sustainable hydrogen release, and further cooperated with an alarmin protein S100A8/A9 inhibitor, ABR2575 (ABR) for synergistic anti‐inflammation therapy toward sepsis‐induced ALI (**Figure** **1**). In this system, the hydrogen donors, SiH nanosheets (NSs), were encapsulated by poly(lactic‐*co*‐glycolic acid) (PLGA) to form sponge‐like structure, which can efficiently slow down the interaction between SiH NSs with H_2_O and achieved prolonged and constant hydrogen release. The released hydrogen can not only relieve the oxidative injury of lung tissue, but also drive the M2 polarization of macrophages, and thus down‐regulate the level of pro‐inflammatory cytokines and inflammatory tissue injury. Besides, the ABR was also encapsulated in the nanosponge to block the activation and infiltration of neutrophils by the interference in NLRP3/IL‐1β signaling pathway. Once intratracheally injected, the nanocomposites arrived at the inflammatory lung area and constantly release hydrogen and ABR molecules, and thus alleviate the in situ inflammatory impairment via multiple pathways, including antioxidation, polarization regulation of macrophages, and inhibition of neutrophils. Meanwhile, the SiH/ABR@PLGA also alleviated the systemic inflammation via the alveola‐circulation communications, which was mainly manifested by the significant inhibition of the activation of global neutrophils and pro‐inflammatory monocytes, as well as the production of pro‐inflammatory cytokines. The sequencing study further highlighted the combination of the anti‐oxidation effects and the inhibition of neutrophil activation of SiH/ABR@PLGA. A variety of molecular and pathological assays supported the SiH/ABR@PLGA‐mediated synergistic roles in protecting against the sepsis‐induced injuries, offering great potential in clinical inventions.

## 2 Results and Discussion

### 2.1 Preparation and Characterization of SiH/ABR@PLGA

**Figure 2:**
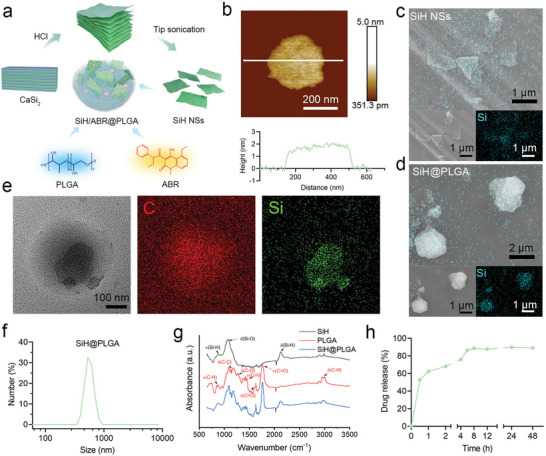
Synthesis and characterizations of SiH/ABR@PLGA. a) Synthetic route and structure of SiH/ABR@PLGA nanosponge. b) AFM image of SiH NS and thicknesses of the representative nanosheet. SEM photographs and related element scanning results of c) SiH NSs and d) SiH@PLGA. e) TEM photograph and element mapping results of SiH@PLGA. Scale bar = 100 nm. f) DLS results of SiH@PLGA. g) FTIR spectra of SiH, PLGA, and SiH@PLGA. h) Drug release profile of ABR from SiH/ABR@PLGA in PBS under 37 °C.

The ultrathin SiH nanosheets were prepared by a one‐step wet‐chemical exfoliation procedure to serve as the hydrogen donor, which can react with water and generate hydrogen under mild conditions without the production of toxic byproducts, holding great potential for in vivo hydrogen therapy. Then, the SiH NSs were encapsulated together with ABR into the core of PLGA nanosponge to obtain SiH/ABR@PLGA. To be specific, SiH sheets were synthesized via the topotactic deintercalation of Ca^2+^ from the layered Zintl‐phase of CaSi_2_ lattice by reaction in hydrochloric acid (HCl) at −20 °C. Then tip sonication was subjected to the SiH sheets to form the nanosized SiH nanosheets (**Figure** **2**a). The successful preparation of SiH NSs was demonstrated by atomic force microscopy (AFM) image, and the SiH NS exhibits an average thickness of approximately 1.73 nm (Figure 2b). Then, the morphology and elemental composition of SiH NSs (Figure 2c) and SiH@PLGA (Figure 2d) were investigated by scanning electron microscopy (SEM). As expected, the distribution of Si element can be observed in both SiH NSs and SiH@PLGA, indicating the successful encapsulation of SiH NSs by PLGA. Further, the structure SiH@PLGA was carefully observed by the transmission electron microscopy (TEM). As shown in Figure 2e, the SiH@PLGA showed a spherical shell which is attributed to the formation of PLGA‐based nanosponge, and Si‐containing nanosheets in the core, demonstrating the SiH NSs were well packaged by the PLGA. Then, the size of SiH@PLGA was demonstrated to be ∼ 568 nm by dynamic light scattering (DLS) (Figure 2f). Further, the presence of SiH and PLGA was also demonstrated by Fourier transform infrared (FTIR) spectra (Figure 2g). To be specific, the peaks at 2116.4 nm and 875.5 nm indicated the presence of Si─H bonds, the peak at 1094.8 nm can be assigned to the Si–O from SiH NSs, the peaks at 1759.5 nm and 1179.4 nm are assigned to the C═O and C─C bonds, respectively, while the existence of C─H bonds can be demonstrated by the peaks ranged from 1348 nm to 1460 nm, which are well matched with the spectra of SiH and PLGA. Based on the successful preparation of SiH@PLGA, ABR, a specific S100A8/A9 inhibitor, was then loaded into the nanosponge to achieve synergistic therapy against ALI. Benefiting from the hydrophobic property of ABR, a high encapsulating efficiency (EE) of 91.7% was achieved, and the drug loading coefficient (DL) was calculated to be 9.1%. The SiH/ABR@PLGA showed similar morphology with SiH@PLGA (Figure S1, Supporting Information) and a slightly increased size (Figure S2, Supporting Information) than SiH@PLGA due to the addition of ABR. Besides, both SiH@PLGA and SiH/ABR@PLGA exhibited negative surface charges (Figure S3, Supporting Information), ensuring their biocompatibility for biomedical use. Next, we investigated the drug release profile of ABR in biological condition by high performance liquid chromatography (HPLC). As shown in Figure 2h, the nanosponge can promise the sustained release of ABR during 12 h, promising the long‐term therapeutic effect in vivo.

### 2.2 Hydrogen Release and ROS Scavenging Capabilities of SiH@PLGA

**Figure 3:**
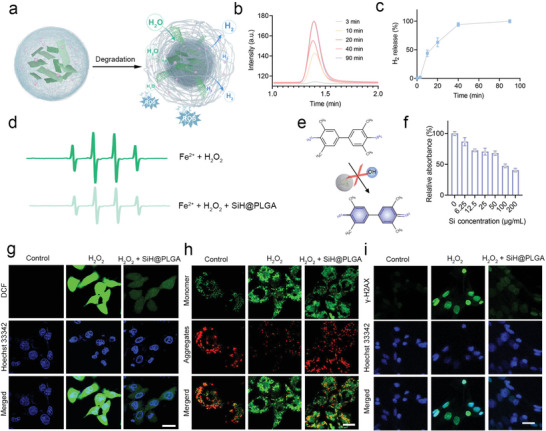
The radical‐scavenging capability of SiH/ABR@PLGA in vitro. a) Mechanism schematics of degradation, hydrogen generation, and ABR release of SiH/ABR@PLGA. b) Gas chromatograms of H_2_ generated by SiH@PLGA in PBS different time periods post‐addition. c) Time‐dependent H_2_ generations of SiH@PLGA suspended in PBS, as measured by gas chromatograph. d) ESR spectra of ·OH generated by Fenton reaction with or without the addition of SiH@PLGA. e) Mechanism of ·OH scavenging detection via TMB chromogenic reaction. f) Relative absorbance of TMB after incubated with Fe^2+^ and H_2_O_2_ in the presence of SiH@PLGA with different Si concentration. g) ROS levels of cells treated with or without SiH@PLGA in the presence of H_2_O_2_ (Si concentration: 20 µg mL^─1^). The cells were stained by DCFH‐DA to indicate the ROS levels. Scale bar = 20 µm. h) Mitochondrial membrane potentials of cells treated with or without SiH@PLGA in the presence of H_2_O_2_ (Si concentration: 20 µg mL^─1^). The cells were stained by JC‐1 to indicate ROS mitochondrial membrane potentials. scale bar = 20 µm. i) DNA impairment indicated by γ‐H2AX levels of cells treated with or without SiH@PLGA in the presence of H_2_O_2_ (Si concentration: 20 µg mL^─1^), scale bar = 50 µm.

We next demonstrated the prolonged release of hydrogen in biological condition. In our design, the SiH NSs were protected by the sponge‐like PLGA, which could limit the contact of SiH NSs with H_2_O, and thus slow down the degradation and hydrogen generation triggered by the reaction between SiH NSs and H_2_O (**Figure** **3**a). As shown in Figure 3b,c, SiH@PLGA nanosponge showed a prolonged hydrogen release profile and can generate hydrogen at a constant speed in 40 min, promising the in situ continuous hydrogen supply at target tissues or organs. To be noted, although the hydrogen generation behavior can facilitate the release of ABR in 1 h, the structure of SiH/ABR@PLGA is not significantly altered (Figure S4, Supporting Information), thus ensuring the sustained release of ABR (Figure 2h). Hydrogen has been largely reported as a therapeutic gas for multiple disease treatment, especially in inflammation‐related diseases for its superior capability for anti‐oxidation and anti‐inflammation. Thus, we first evaluate the ·OH elimination efficiency of SiH@PLGA to demonstrate its anti‐oxidative ability. As reflected by the electron spin resonance (ESR) spectra, with the addition of SiH@PLGA, the peak of ·OH experienced a sharp decrease, indicating the efficient removal of ·OH by SiH@PLGA (Figure 3d). The chromogenic reaction of 3,3′,5,5′‐tetramethylbenzidine (TMB) is also employed to quantify the elimination of ·OH by SiH@PLGA (Figure 3e). As shown in Figure 3f, the SiH@PLGA can obviously remove the ·OH at the SiH concentration of 6.25 µg mL^─1^ and exhibits over 50% inhibition when the concentration reaches 100 µg/mL.

### 2.3 Protective Effect of SiH@PLGA on Lung Epithelial Cells Against Mitochondrial and DNA Injury in Vitro

Based on the above demonstrated ability of prolonged hydrogen release and effective ROS scavenging, we speculated that the SiH@PLGA could protect the lung epithelial cells from oxidative stress injury. Therefore, H_2_O_2_ was added to the lung epithelial cell medium for 2 h to mimic ALI‐induced oxidative stress injury. As excepted, the H_2_O_2_ sharply increased the FL signals of DCF (Figure 3g), which indicates the level of intracellular ROS. The SiH@PLGA remarkably eliminated the intracellular ROS. Meanwhile, H_2_O_2_ also significantly induced the mitochondrial injury (Figure 3h, increased JC‐1 monomer/JC‐1 aggregates (green/red)) and DNA damage (Figure 3i, increased expression of γ‐H2AX, green) of lung epithelial cells. Accordingly, SiH@PLGA significantly alleviated the H_2_O_2_‐induced mitochondrial and DNA injury (Figure 3h,i). Meanwhile, it should be noted that SiH/ABR@PLGA, but not ABR@PLGA, exhibited consistent effects in reducing ROS level, mitochondrial injury and DNA damage (Figure S5, Supporting Information), indicating the protective effects on epithelial cells are attributed to the potent effect of SiH in eliminating intracellular ROS. In contrast, the ABR@PLGA shows only limited effects on H_2_O_2_‐induced damage, suggesting that ABR plays a negligible role in ROS scavenging on epithelial cells in vitro. Furthermore, SiH/ABR@PLGA and its components show negligible toxicity towards MLE‐12 cells (Figure S6, Supporting Information), which inspires us to explore the in vivo applications of SiH/ABR@PLGA.

### 2.4 The Regulation Effect of SiH/ABR@PLGA on Macrophage Polarization to Reduce the Inflammation

**Figure 4:**
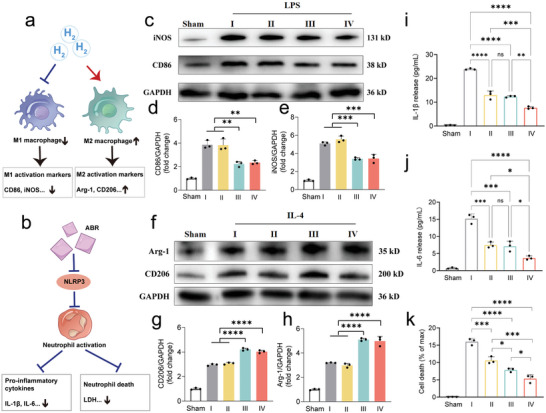
SiH/ABR@PLGA regulates the polarization of macrophages and reduces the activation of neutrophils in vitro. a) Mechanism schematics of the regulation of molecular hydrogen on macrophage polarization. b) Mechanism schematics of the regulation of ABR on neutrophil activation. c–e) The representative western blotting images and quantification of M1 activation markers in the sham, LPS‐treated, LPS‐ and nanocomplexes‐treated macrophages. f–h) The representative western blotting images and quantification of M2 activation markers in the sham, IL‐4‐treated, IL‐4‐ and nanocomplexes‐treated macrophages. i–k) The quantification of the release of IL‐1β, IL‐6 and cell death in the sham, activated, and nanocomplexes‐treated activated neutrophils. The statistical differences were analyzed with Kruskal‐Walli's test followed by a Dunn's multiple comparisons. Data are represented as mean ± SD. * indicted *p* <0.05, ** indicted *p* <0.01, *** indicted *p* <0.001, and **** indicted *p* <0.0001. I‐PBS‐treated activated cells; II‐ABR@PLGA‐treated activated cells; III‐SiH@PLGA‐treated activated cells; IV‐SiH/ABR@PLGA‐treated activated cells.

The mechanisms of SiH/ABR@PLGA in modulating the macrophage polarization and neutrophil activation in ALI were then investigated (**Figure** **4**a,b). In addition to neutrophils, macrophages are another key inflammatory cell in ALI, which massively infiltrate the inflammatory alveoli, transform into M1‐dominated polarization (pro‐inflammatory) and release pro‐inflammatory factors.^[^ ^13^ ^]^ Therefore, inhibition of the M1 polarization or promotion of the M2 polarization (anti‐inflammatory) is considered to be effective to interfere with ALI.^[^ ^14^ ^]^ Previous studies have shown that the molecule hydrogen plays an anti‐inflammatory role by regulating the macrophage polarization.^[^ ^15^ ^]^ Thus, we further evaluated the influence of ABR@PLGA, SiH@PLGA and SiH/ABR@PLGA on macrophage polarization. Lipopolysaccharide (LPS) of 500 ng mL^−1^ or 10 ng mL^−1^ IL‐4 was used to induce the M1 or M2 polarization, respectively. Consistent with the previous investigations, LPS significantly promoted the expression of M1 markers (iNOS and CD86), and IL‐4 could induce the M2 polarization (increased expression of Arg‐1 and CD206). Furthermore, the treatment of SiH/ABR@PLGA or SiH@PLGA, rather than ABR@PLGA, exhibits significant inhibition on M1 polarization (Figure 4c–e), as well as the significant promotion on M2 polarization (Figure 4f–h). The flow cytometry analysis indicated the consistent effects of SiH/ABR@PLGA on macrophage polarization (Figure S7, Supporting Information). These results suggested that the SiH exerts the anti‐inflammatory and protective effects by regulating macrophage polarization in vitro.

### 2.5 The Inhibition Effect of SiH/ABR@PLGA on Neutrophils Activation and Death

Neutrophils play the most critical roles in ALI inflammation,^[^ ^2^ ^]^ so we equipped our nanoparticles with ABR, a specific inhibitor of neutrophils,^[^ ^3c^ ^]^ to alleviate the neutrophils activation and inflammation. Thus, we further performed the in vitro investigations to verify the inhibitory effect of SiH/ABR@PLGA on neutrophil. The stimulated LPS‐primed neutrophils released significantly increased IL‐1β and IL‐6 (Figure 4i,j), and the increased concentration of LDH indicated the significant cell death upon stimulation (Figure 4k). Moreover, the treatment of ABR@PLGA, SiH@PLGA and SiH/ABR@PLGA all displayed significant restoration effects on neutrophils activation and death. The western blotting (WB) analysis also indicated the significant inhibition of TLR4 and NLRP3 inflammasome expression in activated neutrophils under the treatments of ABR@PLGA, SiH@PLGA and SiH/ABR@PLGA (Figure S8, Supporting Information). In particular, SiH/ABR@PLGA‐treated cells showed significant better recovery as compared with ABR@PLGA and SiH@PLGA groups (Figure 4i–k). The above results suggest that both SiH and ABR inhibited neutrophil activation, both of which contributed to the protective effects of SiH/ABR@PLGA on ALI‐induced injury. In summary, the present data confirm that SiH/ABR@PLGA can eliminate ROS, protect epithelial cells and inhibit neutrophil and macrophage activation in vitro. These multiple functions support the therapeutic effect on ALI, further encouraging us to conduct in vivo experiments.

### 2.6 Lung Injury Restoration of SiH/ABR@PLGA in ALI Mouse Model

**Figure 5:**
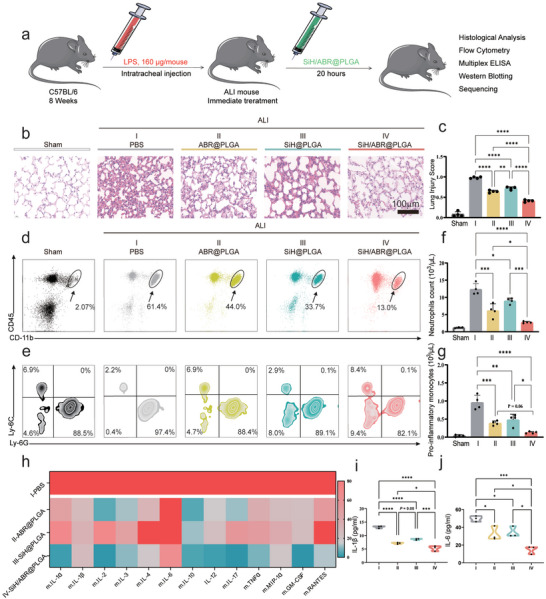
SiH/ABR@PLGA relieves the lung injury and systematic inflammation in ALI mice. a) Flow chart of animal experiments. (b) The representative images of H&E‐stained lung sections in sham, ALI, and nanocomplexes‐treated ALI mice; the scale bar = 100 µm. (c) The total lung injury score based on the histological images. (d–g) The representative flow cytometric images and the quantification of neutrophils and pro‐inflammatory monocytes in the blood. (h) The heat map of multiple inflammatory cytokines in the serum of ALI and nanocomplexes‐treated ALI mice (The heat map showed the normalized level for each cytokine, and the raw data can be detected in Figure S11, Supporting Information). (i–j) The quantification of IL‐1β and IL‐6 revealed by the multiple ELISA. The statistical differences were analyzed with Kruskal‐Walli's test followed by a Dunn's multiple comparisons. Data are represented as mean±SD. * indicted *p* <0.05, ** indicted *p* <0.01, *** indicted *p* <0.001, and **** indicted *p* <0.0001. I‐ALI + PBS‐treated group; II‐ALI + ABR@PLGA‐treated group; III‐ALI + SiH@PLGA‐treated group; IV‐ALI + SiH/ABR@PLGA‐treated group.

To evaluate the in vivo therapeutic effect of SiH/ABR@PLGA, the ALI model was induced by the intratracheal injection of LPS. The sham mouse received the injection of equivalent volume of PBS. The ALI mice were treated with ABR@PLGA, SiH@PLGA and SiH/ABR@PLGA to assess the in vivo therapeutic efficacy. 20 h after the injection, the mice were sacrificed, and the left lung lobe was harvested for the histological assessment of lung injury (**Figure** **5**a). As shown in Figure 5b,c; Figure S9 (Supporting Information), the injection of LPS led to the remarkable lung injury, as indicated by the significant score increases of all the five features. Meanwhile, the treatment of ABR@PLGA, SiH@PLGA and SiH/ABR@PLGA exhibited significant decreases in all the five features (all *p* < 0.05), suggesting the restoration of lung injury. Particularly, the SiH/ABR@PLGA‐treated group exhibited significant therapeutic improvements, as compared with the ABR@PLGA and SiH@PLGA groups (all *p* < 0.05, Figure 5c; Figure S9, Supporting Information).

### 2.7 Suppression of ALI‐Induced Systemic Inflammation by the Treatment of SiH/ABR@PLGA

Systemic inflammation represents another essential process in ALI pathophysiology, mainly manifested by the prominent activation of inflammatory cells and extensive release of inflammatory cytokines.^[^ ^13^, ^14^ ^]^ Thus, we utilized the flow cytometry of peripheral blood (gating strategy seen in Figure S10, Supporting Information) to explore the influences of nanocomplexes on activation of inflammatory cells, as well as the multiplex ELISA of serum to assess the influence on cytokines. As shown in Figure 5d–f, the ALI mice showed significantly increased neutrophils in the blood, which was significantly decreased by the treatment of ABR@PLGA, SiH@PLGA and SiH/ABR@PLGA (all *p* < 0.05). Furthermore, the SiH/ABR@PLGA‐treated group displayed the significantly less activation, as compared with the ABR@PLGA‐ and SiH@PLGA‐treated groups (*p* < 0.05, < 0.001, respectively, Figure 5g). Meanwhile, the count of pro‐inflammatory monocytes (Ly6C^−hi^) displayed the consistent trend of change under the treatments of nanocomplexes (all *p* < 0.05), with the exception that the difference between the ABR@PLGA and SiH/ABR@PLGA group failed to reach the significant level (P = 0.06, Figure 5f).

As detected by the multiplex enzyme‐linked immunosorbent assay (ELISA) (Figure 5h), the inflammatory cytokines in the serum were significantly reduced in the ABR@PLGA, SiH@PLGA and SiH/ABR@PLGA groups (all *p* < 0.05), as compared to the ALI group. SiH/ABR@PLGA‐treated mice displayed further inhibited expression of cytokines when comparing to ABR@PLGA‐ and SiH@PLGA‐treated groups (*p* < 0.05, seen in Figure 5h—j; Figure S11, Supporting Information). Taken together, the data indicated the remarkable anti‐inflammatory effects of SiH/ABR@PLGA when comparing to the single treatment, by effectively inhibiting the activation of pro‐inflammatory cells and the expression of inflammatory cytokines in the blood.

### 2.8 Inhibition Effect of SiH/ABR@PLGA on Local Inflammation in ALI Mice

**Figure 6:**
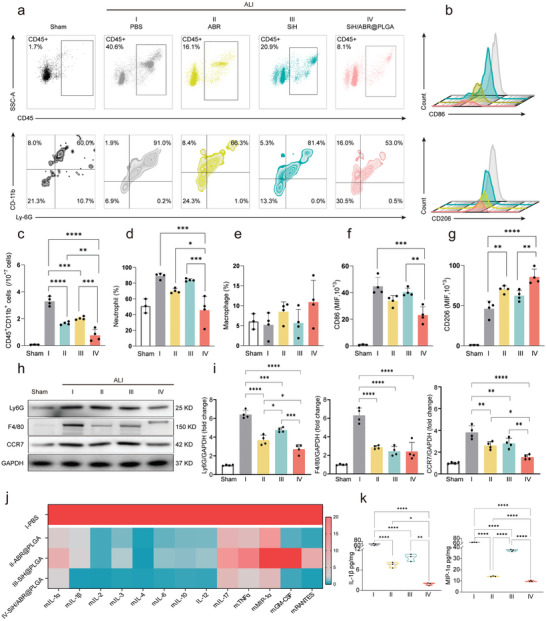
SiH/ABR@PLGA inhibits local inflammation in ALI mice. a) The representative flow cytometric images of CD45^+^ inflammatory cells, neutrophils, and macrophages in the BALF in sham, ALI, and nanocomplexes‐treated ALI mice. b) The representative flow cytometric images of CD86 and CD206 MFI of macrophages in the BALF. c–e) The quantification of CD45^+^CD11b^+^ inflammatory cells, neutrophils, and macrophages in the BALF. f,g) The quantification of CD86 and CD206 MFI of macrophages in the BALF (The proportion of M1 and M2 activated macrophages was shown in Figure S13, Supporting Information). h,i) The representative western blotting images and quantification of markers of inflammatory cells in the lung tissue. j) The heat map of multiple inflammatory cytokines in the lung tissue of ALI and nanocomplexes‐treated ALI mice (the normalized level for each cytokine was illustrated in the heat map, and the raw data can be detected in Figure S17, Supporting Information). k) The quantification of IL‐1β and MIP‐1a was revealed by the multiple ELISA. The statistical differences were analyzed with Kruskal‐Walli's test followed by a Dunn's multiple comparisons. Data are represented as mean±SD. * indicted *p* <0.05, ** indicted *p* <0.01, *** indicted *p* <0.001, and **** indicted *p* <0.0001. I‐ALI+PBS‐treated group; II‐ALI+ABR@PLGA‐treated group; III‐ALI+SiH@PLGA‐treated group; IV‐ALI+SiH/ABR@PLGA‐treated group.

To further evaluate the changes of pulmonary inflammation, we comprehensively evaluated the infiltration of inflammatory cells and the expression of inflammatory cytokines in BALF and lung tissue. Our data implied that the nanocomplexes significantly reduced the frequency of CD45^+^CD11b^+^ inflammatory cells infiltrating in the BALF (gating strategy seen in Figure S12, Supporting Information), which was mainly attributed to the inhibition of neutrophils (**Figure** **6**a,c,d). Regarding the macrophages, the nanocomplexes failed to have a significant influence on the cell counts (Figure 6e), but significantly promoted the degree of M2 polarization (as detected by both the increased MFI of CD206 and increased proportion of M2‐activated macrophages). Furthermore, the M1‐activation of macrophages was also significantly inhibited by the nanocomplexes (Figure 6b,f,g; Figure S13, Supporting Information).

Similarly, in the lung tissue, the SiH/ABR@PLGA significantly inhibited the infiltration of inflammatory cells, as detected by both the immunofluorescence analyses (Figure S14, Supporting Information) and WB (Figure 6h,i), the expressions of inflammatory cell markers were significantly reduced upon the treatment of nanocomplexes. Particularly, the myeloperoxidase (MPO) staining indicated the inhibitory effects of the nanocomplexes on the neutrophil recruitment and activations in ALI mice (Figure S15, Supporting Information). And the TLR4‐NLRP3 Inflammasome signaling was also significantly inhibited upon the treatments of nanocomplexes (Figure S16, Supporting Information). Further, among all the nanocomplexes, the SiH/ABR@PLGA displayed the most potent inhibition effect (Seen in Figure 6i). Meanwhile, the expressions of inflammatory cytokines showed similar changes (Figure 6j; Figures S17,S18, Supporting Information). All the above treatments significantly reduced the expression of cytokines, and the most potent therapeutic effects were found in the SiH/ABR@PLGA‐treated group. Regarding the differences between ABR@PLGA and SiH@PLGA, the ABR@PLGA‐treated group generally showed a stronger inhibitory effect, while the differences between the two groups sometimes did not reach the level of significance (Figure 6j,k; Figure S17, Supporting Information). In summary, the ABR@PLGA, SiH@PLGA and SiH/ABR@PLGA all played powerful inhibitory effects against the local lung inflammation in ALI mice, and similarly, the most prominent anti‐inflammatory effects were found in the SiH/ABR@PLGA‐treated group.

### 2.9 Molecular Mechanism Investigations of SiH/ABR@PLGA by the Transcriptomic Analysis

**Figure 7:**
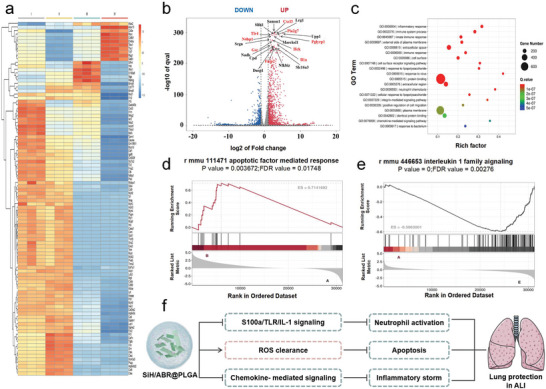
Transcriptome profiles of the inflammatory cells in the BALF of the treated ALI mice reveal the potential therapeutic mechanism. a) The alterations of the inflammatory cell transcription in the nanocomplexes‐treated ALI mice. I‐ ALI+PBS‐treated group; II‐ ALI+ABR@PLGA‐treated group; III‐ ALI+SiH@PLGA‐treated group; IV‐ ALI+SiH/ABR/@PLGA‐treated group. b) The up‐ and down‐ regulated DEGs in the ALI and SiH/ABR@PLGA‐treated ALI mice. c) The GO term shows the most down‐regulated pathways under the treatment of SiH/ABR@PLGA in ALI mice. d) The GSEA highlights the significant inhibition of apoptotic response in the SiH/ABR@PLGA‐treated ALI mice. e) The GSEA highlights the significant inhibition of IL‐1 signaling in the SiH/ABR@PLGA‐treated ALI mice. f) A brief summary of the mechanisms underlying the lung protections of SiH/ABR@PLGA revealed by the transcriptomic analysis.

To reveal the mechanisms underlying the protective effects of SiH/ABR@PLGA on ALI model, the RNA sequencing of inflammatory cells in the BALF was utilized. The treatments of the nanocomplexes led to significant alterations in inflammatory cell transcription in the ALI mice (**Figure** **7**a). Of note, compared to the SiH/ABR@PLGA‐treated mice, totally 2552 DEGs were found in the ALI group (LPS‐treated), 1396 of which were up‐regulated and 1156 of which were down‐regulated differentially expressed genes (DEGs). Furthermore, a substantial portion of the top 20 DEGs were involved in the regulation of oxidative stress and inflammatory cell activation (highlighted by red, seen in Figure 7b). For further reveal the anti‐inflammatory mechanisms by SiH/ABR@PLGA on ALI, the Gene Ontology (GO) and Gene Scores Enrichment Analysis (GSEA) enrichment analysis were performed (Figure 7c–e). As expected, the DEGs between the ALI and SiH/ABR@PLGA‐treated group were significantly enriched in the pathways related to immune regulations, including the inflammatory/immune response pathways, response to LPS pathways, neutrophil chemotaxis pathways, chemotaxis‐mediated signaling pathways (Figure 7c).

As identified by previous investigations, hydrogen produced by SiH is a powerful anti‐apoptotic mediator, and ABR represents an effective inhibitor of neutrophil activation. Consequently, the SiH/ABR@PLGA nanocomplexes may exhibit the strongest therapeutic effect on ALI by combining the anti‐apoptotic and anti‐inflammatory actions of the two. The current sequencing data supported the above hypothesis, as the GSEA analysis illustrated that the SiH/ABR@PLGA played more powerful anti‐apoptotic roles as comparing to the ABR@PLGA (Seen in Figure 7d). Meanwhile, a stronger inhibition of IL‐1 signaling was detected in SiH/ABR@PLGA, as compared to SiH@PLGA (Figure 7e). Collectively, the sequencing analysis helped to reveal the mechanism by which the SiH/ABR@PLGA down‐regulated the signaling pathways related to the neutrophilic activation, apoptosis, and chemokines‐mediated inflammatory response, to protect mice after ALI against the systemic and local inflammatory injuries (Figure 7f).

## 3 Conclusion

To conclude, we have developed an immunoregulatory SiH/ABR@PLGA nanocomplexes for the treatment of ALI. SiH/ABR@PLGA was found to effectively reduce the oxidative stress by generating H_2_ to scavenge ·OH. Further, the established nanocomplexes possessed the specific regulatory activity of neutrophil activation by blocking S100A8/A9‐TLRs‐IL‐1 signaling, thus inhibiting the inflammatory storm post ALI. Specifically, SiH/ABR@PLGA dramatically inhibited the frequencies of neutrophils and pro‐inflammatory monocytes and macrophages, while increased the proportion of anti‐inflammatory M2 macrophages in both the systemic and local inflammation. The combination of antioxidant SiH and anti‐inflammatory ABR significantly improved the therapeutic effect in alleviating lung injury, which was further supported by the transcriptome profiles. The present study provides a novel strategy to integrate nanomedicine therapy with immunotherapy for durable, effective, and specific treatment of ALI, offering potential solutions for the in vivo delivery of chemically active therapeutics. In follow‐up studies, we attempt to improve the SiH/ABR@PLGA to achieve a sustained release of drugs, so as to realize the long‐term inhibition of inflammatory injury. Furthermore, the timing of SiH/ABR@PLGA administration could also be optimized, which may represent one way to further improve the therapeutic efficacy.

## 4 Experimental Section

### Chemical Reagents

ABR215757, the S100A8/A9 inhibitor, was purchased from the MedChemExpress (NJ, USA, Cat. No.: HY‐100442, purity: 99.90%). Calcium silicide (CaSi_2_, tech‐95, 95%, powder, Gelest) was purchased from Alfa Aesar (MA, USA). Hydrochloric acid (HCl, 35–37%) and acetone (AR, ≥99.5%) were purchased from Sinopharm Chemical Reagent Co., Ltd. (Shanghai, China). Ethanol (AR, ≥99.5%) was bought from Shanghai Lingfeng Reagent Chemical Co., Ltd. (Shanghai, China). Poly (lactic‐*co*‐glycolic acid) (PLGA) was purchased from Aladdin Chemistry Co., Ltd. (Shanghai, China). 5,5‐dimethyl‐1‐pyrroline *N*‐oxide (DMPO, ≥99%) was purchased from Dojindo China Co., Ltd.

### Preparation and Characterization of SiH NSs, SiH@PLGA, and SiH/ABR@PLGA

To synthesize SiH NSs, 1 g CaSi_2_ was dispersed in 100 mL of precooled HCl and kept stirring for 7 days at −20 °C under argon protection. Then, the SiH sheets were centrifuged (12 000 rpm, 10 min) and sequentially washed with ethanol for 3 times. The product was dispersed in 100 mL of ethanol and subjected to 8 h tip sonication (600 W, on: 2 s, off: 4 s) under ice bath to obtain SiH NSs.

To prepare SiH@PLGA and SiH/ABR@PLGA, 2 mg SiH NSs and 4 mg PLGA were first dispersed in acetone, respectively, and ABR was dissolved in DMSO (50 mg mL^−1^). Then the SiH NSs, PLGA, and ABR were mixed and added into HCl solution (pH = 4.0) drop wise and kept stirring for 20 min. Then the acetone was removed by vacuum rotary evaporation, and the SiH/ABR@PLGA were centrifuged and washed with H_2_O for 3 times to remove DMSO and the unloaded drugs. The SiH@PLGA was obtained via the similar method without adding ABR.

Atomic force microscope was applied to measure the thickness of nanosheets by Bruker Dimension ICON. The quantitative elemental analysis was detected by inductively coupled plasma‐optical emission spectrometry (ICP‐OES, Agilent 700 Series, Agilent Technologies, US). TEM (JEM‐2100F, JEOL) and SEM (S‐4700, Hitachi) were applied to evaluate the morphology of SiH nanosheets and SiH@PLGA nanosponges. The hydrodynamic particle size and zeta potential of SiH@PLGA and SiH/ABR@PLGA were measured by a zetasizer (Nano AS90, Malvern Instrument). The infrared spectra of SiH, PLGA, and SiH@PLGA were collected using a Thermo Scientific Nicolet iS10 Fourier transform infrared (FTIR) spectrometer to demonstrate the successful preparation of SiH@PLGA.The as‐prepared SiH/ABR@PLGA and SiH@PLGA was stored at −20 °C after lyophilization.

The encapsulation efficiency (EE%) and drug loading coefficient (DL%) of SiH/ABR@PLGA were calculated by the following equations:

(1) $$\begin{array}{ccc} \mathrm{EE}\% & = & \mathrm{Weight}\;\mathrm{of}\;\mathrm{loaded}\;\mathrm{ABR}\;\mathrm{in}\;\mathrm{SiH}/\mathrm{ABR}@\mathrm{PLGA}\\ & & /\mathrm{Weight}\;\mathrm{of}\;\mathrm{the}\;\mathrm{feeding}\;\mathrm{ABR}\times 100\% \end{array}$$

(2) $$\begin{array}{ccc} \mathrm{DL}\% & = & \mathrm{Weight}\;\mathrm{of}\;\mathrm{loaded}\;\mathrm{ABR}\;\mathrm{in}\;\mathrm{SiH}/\mathrm{ABR}@\mathrm{PLGA}\\ & & /\mathrm{Weight}\;\mathrm{of}\;\mathrm{SiH}/\mathrm{ABR}@\mathrm{PLGA}\times 100\% \end{array}$$

### Drug Release of ABR from SiH/ABR@PLGA

The release behavior of SiH/ABR@PLGA was monitored as follows. Briefly, 5 mL of SiH/ABR@PLGA suspension was introduced into a dialysis bag (MWCO = 2 kDa), and the dialysis bag was incubated in 50 mL PBS solution containing 0.1% w/v Tween 80 under shaking at 37 °C. Then, 1 mL of the medium outside the dialysis bag was withdrawn at different incubation time points for quantification and replaced with an equal volume of fresh medium. The concentration of ABR in medium was determined by Agilent 1260 Infinity II high‐performance liquid chromatography (HPLC) system.

### Hydrogen Generation and Antioxidation Capability Assessment of SiH@PLGA

To measure the generation of H_2_, SiH NSs and SiH@PLGA were first dispersed in deionized water at a Si concentration of 2 mg mL^−1^, respectively. Then, 500 µL of SiH or SiH@PLGA dispersion was added to a sealed 10 mL round‐bottom flask containing 2 mL of PBS, which was filled with nitrogen. Next, 1 mL of gas in the flask was extracted using a syringe at different time points and detected by gas chromatography (GC2010, Shimadzu). The ·OH Scavenging capacity of SiH@PLGA was investigated by chromogenic reaction of TMB with ·OH and ESR test. To be specific, the FeSO_4_ (1 mM) and H_2_O_2_ (2 mM) were mixed in PBS (pH = 6.0), followed by the addition of SiH@PLGA. After 8 min reaction, the TMB was added, and absorbance at 650 nm was measured by a microplate reader (BioTek Instruments, Winooski, VT). To assess the ESR test, FeSO_4_ (1 mM) and H_2_O_2_ (2 mM) were mixed together in PBS (pH = 6.0) to generate ·OH via Fenton reaction. The SiH@PLGA was added to the reaction to scavenge the ·OH, and the H_2_O with same volume was added to the mixture in control group. DMPO was added at the beginning to capture the ·OH and form DMPO‐OH adducts. The ·OH scavenging efficiency was estimated according to the decrease of peak intensity DMPO‐OH according to the ESR spectra.

### In Vitro Experiments

*Cell Models*: The neutrophils were obtained from the mice bone marrow via the Mouse Neutrophil Enrichment Kit (STEMCELL, 19 762) according to the protocol. The lung epithelial cell lines (MLE‐12, STCC20008G) and macrophage cell lines (RAW264.7, STCC20020P) were purchased from the Servicebio company. To mimic the ALI‐induced oxidative injury, 800 µM H_2_O_2_ was added into the medium of epithelial cell for 2 h. To assess the polarization of macrophages, LPS (500 ng mL^−1^) or IL‐4 (10 ng mL^−1^) were added to the medium for 12 h to induce the M1 and M2 polarization, respectively. To mimic the ALI‐induced canonical inflammasome activation of neutrophils, E‐selectin (1 µg mL^−1^) was utilized to stimulate the LPS‐primed (1 µg mL, 2.5 h) neutrophils for 15 minutes as previously described ^33^. To evaluate the cell death of neutrophils, E‐selectin (1 µg mL^−1^) was used to stimulate the neutrophils for 30 min. To assess the safety of SiH/ABR@PLGA, the lung epithelial cells were incubated with SiH, SiH@PLGA, ABR, PLGA, and SiH/ABR@PLGA (Si: 0, 25, 50, 75, 100 µg mL^−1^) for 24 h and the cell viability was investigated by Cell Counting Kit‐8 (CCK‐8) assay. The concentrations indicate the Si concentrations in SiH/ABR@PLGA group, and the concentrations of other components were kept consistent with those in SiH/ABR@PLGA group.

### Detection of Mitochondrial Function and Intracellular ROS in Lung Epithelial Cells

The antioxidant effect of SiH/ABR@PLGA were measured by JC‐1 staining. Briefly, the lung epithelial cells were treated with H_2_O_2_ (800 µM in culture medium without FBS) with or without SiH@PLGA (Si concentration: 20 µg mL^−1^) 4 h. The cells were then stained with JC‐1 or DCFH‐DA to indicate the mitochondrial function or intracellular ROS level, respectively. Before confocal imaging, the cells were stained with Hoechst 33 342 for 10 min and washed with PBS for 3 times.

### Assessment of DNA Injury in Lung Epithelial Cells

The injury of DNA was measured by γ‐H2AX staining (Beyotime, C2035S) in another parallel experiment. After the treatments mentioned above, the epithelial cells were fixed and blocked, and then incubated with anti‐γ‐H2AX primary antibody overnight. After washing, the cells were incubated with the secondary antibody at room temperature for 1 h and then photographed with a confocal microscope (ZEISS, LSM710).

### Measurement of the Polarization of Macrophages

The polarization of macrophages was measured by WB. The macrophages were incubated in DMEM containing PBS, ABR@PLGA (15 µg mL^−1^ ABR), SiH@PLGA (50 µg mL^−1^ SiH) or SiH/ABR@PLGA (50 µg mL^−1^ SiH and 15 µg mL^−1^ ABR) for 1 h and then LPS or IL‐4 were added to the medium respectively. 12 h later, the cells were harvested and lysed in buffer. The WB was performed as the same protocol with the previous experiments. The antibodies used presently were shown as following: iNOS: Abmart, T55993, 1:500; CD86: Abmart, T55238, 1:1000; Abmart, T55101, 1:1000; CD206: Abmart, TU313804, 1:2000.

### Evaluation the Activation and Cell Death of Neutrophils

The activation of neutrophils was evaluated via ELISA, and the cell death was detected via the LDH release assay. After the incubation with PBS, ABR@PLGA (15 µg mL^−1^ ABR), SiH@PLGA (50 µg mL^−1^ SiH) or SiH/ABR@PLGA (50 µg mL^−1^ SiH and 15 µg mL^−1^ ABR) for 1 h, the neutrophils were stimulated. The supernatants were harvested, and the concentrations of pro‐inflammatory cytokines were measured by ELISA kit (IL‐1β: Invitrogen, BMS6002‐2; IL‐6: Invitrogen, KMC0061). The concentration of LDH were quantified using a LDH Assay (Thermo Fisher, C20301) and the cell death % was calculated as previously described.^[^ ^16^ ^]^

### Mice and Treatments

The adult C57BL/6J mice (male, 8 weeks old) were purchased from the Cyagen Laboratory. The experiments were approved by the Animal Care and Use Committee of Shanghai Chest Hospital (KS(Y)24 069). At operation, the mouse was anesthetized with sodium pentobarbital (80 mg kg^−1^, i.p.), and 160 µg LPS was intratracheally injected to induce the ALI model. Simultaneously, PBS, ABR@PLGA (18 µg ABR per mouse), SiH@PLGA (60 µg SiH per mouse) or SiH/ABR@PLGA (18 µg ABR and 60 µg SiH) was intratracheally injected to assess the in vivo treatment efficiency. The sham mice received the PBS as the vehicle control. The mice were sacrificed 20 hours after the injection, and the BALF, lung tissue and blood were harvested for further investigation. For each group, four mice were included. The left lung lobes were harvested for histological analysis (n = 4), the BALF and peripheral leukocyte were harvested for flow cytometry analysis (n = 4), the right middle and upper lobes and serum were harvested for western blotting and multiplex ELISA (n = 4). Regarding the RNA sequencing, 15 mice were included in each group and their BALF were randomly pooled into 3 samples for further sequencing (n = 3).

### Histological and Immunofluorescence Analyses of Lung Injury and Local Inflammation

For each model, the left lung lobe was dissected and fixed for the tissue section. After the routine process, the lung section (4 µm thick) was stained with hematoxylin‐eosin (H&E) staining to measure the lung injury as previously described.^[^ ^17^ ^]^ Briefly, the five independent features: alveolar neutrophils, interstitial neutrophils, hyaline membranes, proteinaceous debris filling airspaces and alveolar septal thickening were estimated in six distinct areas by two independent investigators in a blinded manner. 0, 1, or 2 were scored for each index based on the severity of injury, and after being differently weighted, the sum of these weighted scores was averaged, leading to a final score between 0 and 1. Furthermore, the aforementioned slides were also stained with the primary antibodies against LY6G (Invitrogen, PA5‐141170, 1:100), F4/80 (Abcam, ab300421, 1:100), CCR7 (Invitrogen, MA5‐31992, 1:200), IL‐1β (Abcam, ab254360, 1:100), IL‐6 (Abcam, ab290735, 1: 100), TNFα (Abmart, PY19810S, 1:500), and MPO (Proteintech, 66177‐1‐Ig, 1:500) to assess the local activation of immune cells and expression of inflammatory cytokines. All the images were analyzed with ImageJ software.

### Flow Cytometry

*BALF*: The BALF was acquired according to the previous investigations.^[^ ^17^, ^18^ ^]^ The inflammatory cells in the BALF were obtained by centrifugation at 350 g for 5 min. After being washed, the cells were resuspended in 50 µL buffer containing the antibodies for Live/Dead marker and surface proteins as described below.

Data were acquired on a NL‐CLC flow cytometer (Cytek Biosciences, Fremont, CA), and the analysis was performed with FlowJo software. After excluding the doublets and dead cells, the neutrophils were identified as CD45^+^, CD11b^+^, Ly6G^+^ cells and the macrophages as CD45^+^, CD11b^+^, Ly6G^−^. To identify the polarization state of macrophages, the mean fluorescence intensity (MFI) of CD86 and CD206 was further evaluated.

*Peripheral Leukocyte*: The peripheral leukocytes were washed and resuspended in 100 µL of FACS buffer containing antibodies against different types of leukocytes. The stained cells were tested on a NL‐CLC flow cytometer, and the data was analyzed via FlowJo software. The CD45^+^, CD11b^+^, Ly6G^+^ cells were identified as neutrophils, while the CD45^+^, CD11b^+^, Ly6G^−^, CD115^+^ cells were identified as monocytes, which were further classified as pro‐inflammatory (Ly6C^−hi^) and anti‐inflammatory (Ly6C^−low^) monocytes.

### Multiplex ELISA of Lung and Serum

0.5 mL whole blood/mouse was taken by cardiac puncture and the serum was acquired by centrifugation (5000 rpm, 10 min). The right lung lobe was dissected for each mouse, and the protein was extracted by the commercial kits according to the manual (Solarbio, BC3710). The obtained serum and lung protein were used for further multiplex ELISA (Boster Bro, MEK1003, 13‐plex) to comprehensively assess the local and systemic inflammatory changes. Further, the concentration of total protein in the lung was assessed by the BCA protein assay kit.

### Western Blotting of Lung Tissue

The protein was extracted from the right lung lobe from each mouse and the protocol followed the routine procedure. The primary antibodies used were shown as following: LY6G (Invitrogen, PA5‐141170, 1:1000), F4/80 (Abcam, ab300421, 1:1000), CCR7 (Invitrogen, MA5‐31992, 1:5000), TLR4 (Proteintech, 19811‐1‐AP, 1:1000) and NLRP3 (Proteintech, 30109‐1‐AP, 1:1000)

### RNA Sequencing of BALF Inflammatory Cells

The BALF were harvested as previously described^[^ ^17^, ^18^ ^]^ and the inflammatory cells were obtained by centrifugation (350 g, 5 min). The total RNA of the cells was extracted by Trizol. The DESeq algorithm was utilized to conduct the differential expression analysis, and the FDR‐corrected *p* < 0.05, log_2_FC > 1 or < −1 was identified for the threshold for DEGs. To further explore the functional and biological implications of the DEGs, the GO and GSEA enrichment analysis were conducted.

### Statistical Analysis

All data were presented as mean ± SD. To compare the differences among three or more groups, the Kruskal‐Wallis (K‐W) test followed by Dunn's multiple comparisons was performed. The P value < 0.05 was considered statistically significant. All analyses were performed via SPSS software.

## Conflict of Interest

The authors declare no conflict of interest.

## Author Contributions

F.S., C.Z., and Q.Z. contributed equally to this work. B.H., H.L., and J.S. designed the experiments; F.S., Y.X.Z., C.Z., Q.Z., Y.S. and S.L. performed the experiments; F.S. and Y.X.Z. carried out the data analysis and wrote the manuscript. H.L. and J.S. supervised the research. All authors proofed and approved the final version of this manuscript for submission.

## Supporting information



Supporting Information

## Data Availability

The data that support the findings of this study are available from the corresponding author upon reasonable request.

## References

[1] a) M. B. Beasley , Mod. Pathol. 2022, 35, 1;34504310

[2] a) G. Renieris , E. Karakike , T. Gkavogianni , D. E. Droggiti , E. Stylianakis , T. Andriopoulou , V. M. Spanou , D. Kafousopoulos , M. G. Netea , J. Eugen‐Olsen , J. Simard , E. J. Giamarellos‐Bourboulis , J. Innate Immun. 2022, 14, 643; 35545011 10.1159/000524560 PMC9801253

[3] a) Y. Sun , B. Hu , G. Stanley , Z. M. Harris , S. Gautam , R. Homer , J. L. Koff , G. Rajagopalan , Am. J. Respir. Cell Mol. Biol. 2023, 68, 75; 36125351 10.1165/rcmb.2022-0117OC PMC9817908

[4] a) Q. Wang , G. Long , H. Luo , X. Zhu , Y. Han , Y. Shang , D. Zhang , R. Gong , Biomed. Pharmacother. 2023, 168, 115674; 37812889 10.1016/j.biopha.2023.115674

[5] a) M. Mohsin , G. Tabassum , S. Ahmad , S. Ali , M. A. Syed , Mitochondrion 2021, 59, 63; 33894359 10.1016/j.mito.2021.04.009

[6] a) W. Ornatowski , Q. Lu , M. Yegambaram , A. E. Garcia , E. A. Zemskov , E. Maltepe , J. R. Fineman , T. Wang , S. M. Black , Redox Biol. 2020, 36, 101679; 32818797 10.1016/j.redox.2020.101679 PMC7451718

[7] I. Ohsawa , M. Ishikawa , K. Takahashi , M. Watanabe , K. Nishimaki , K. Yamagata , K. Katsura , Y. Katayama , S. Asoh , S. Ohta , Nat. Med. 2007, 13, 688. 17486089 10.1038/nm1577

[8] a) H. Dai , Q. Fan , C. Wang , Exploration 2022, 2, 20210157; 37324799 10.1002/EXP.20210157 PMC10191059

[9] a) K. Xie , Y. Yu , Y. Huang , L. Zheng , J. Li , H. Chen , H. Han , L. Hou , G. Gong , G. Wang , Shock 2012, 37, 548; 22508291 10.1097/SHK.0b013e31824ddc81

[10] G. Zhou , E. Goshi , Q. He , Adv. Healthcare Mater. 2019, 8, e1900463. 10.1002/adhm.201900463 31267691

[11] a) Y. Xu , M. Fan , W. Yang , Y. Xiao , L. Zeng , X. Wu , Q. Xu , C. Su , Q. He , Adv. Mater. 2021, 33, 2101455; 10.1002/adma.202101455 34369623

[12] a) W. L. Wan , Y. J. Lin , P. C. Shih , Y. R. Bow , Q. Cui , Y. Chang , W. T. Chia , H. W. Sung , Angew. Chem., Int. Ed. 2018, 57, 9875; 10.1002/anie.201806159 29923670

[13] a) P. Cheng , S. Li , H. Chen , Cells 2021, 10, 436; 33670759 10.3390/cells10020436 PMC7923175

[14] X. Chen , J. Tang , W. Shuai , J. Meng , J. Feng , Z. Han , Inflamm. Res. 2020, 69, 883. 32647933 10.1007/s00011-020-01378-2 PMC7347666

[15] a) T. Itoh , N. Hamada , R. Terazawa , M. Ito , K. Ohno , M. Ichihara , Y. Nozawa , M. Ito , Biochem. Biophys. Res. Commun. 2011, 411, 143; 21723254 10.1016/j.bbrc.2011.06.116

[16] M. Pruenster , R. Immler , J. Roth , T. Kuchler , T. Bromberger , M. Napoli , K. Nussbaumer , I. Rohwedder , L. M. Wackerbarth , C. Piantoni , K. Hennis , D. Fink , S. Kallabis , T. Schroll , S. Masgrau‐Alsina , A. Budke , W. Liu , D. Vestweber , C. Wahl‐Schott , J. Roth , F. Meissner , M. Moser , T. Vogl , V. Hornung , P. Broz , M. Sperandio , Nat. Immunol. 2023, 24, 2021. 37903858 10.1038/s41590-023-01656-1 PMC10681899

[17] a) L. Sun , Y. Liu , X. Liu , R. Wang , J. Gong , A. Saferali , W. Gao , A. Ma , H. Ma , S. E. Turvey , S. Y. Fung , H. Yang , Adv. Sci. 2022, 9, e2104051; 10.1002/advs.202104051 PMC8787384 34816630

[18] Y. Xiong , W. Gao , F. Xia , Y. Sun , L. Sun , L. Wang , S. Ben , S. E. Turvey , H. Yang , Q. Li , Adv. Healthcare Mater. 2018, 7, e1800510. 10.1002/adhm.201800510 30101578

