# Supplementary material for: Multifaceted Immunomodulatory Nanocomplexes Target Neutrophilic‐ROS Inflammation in Acute Lung Injury
Source: Adv Sci (Weinh). 2024 Dec 31;12(8):2411823. doi: 10.1002/advs.202411823 (PMC11848588; doi:10.1002/advs.202411823)
Supplement: Supplementary file 1 — Supporting Information [file ADVS-12-2411823-s001.docx]

**Supporting Information**

**Multifaceted immunomodulatory nanocomplexes target neutrophilic-ROS inflammation in acute lung injury**

*Fan Su*^‡^*, Chong Zhang*^‡^*, Qian-Yun Zhang*^‡^*, Yi Shen, Sai-Qi Li, Jianlin Shi, Ya-Xuan Zhu*, Han Lin*, and Bin He**

^‡^*These authors contributed equally to this work.*

**Additional results and figures.**

**
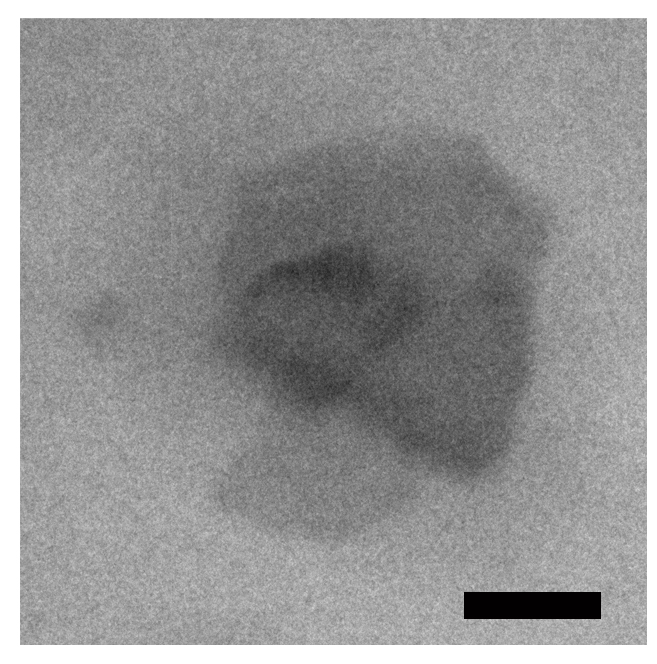
**

**Figure S1.** TEM photograph of SiH/ABR@PLGA.


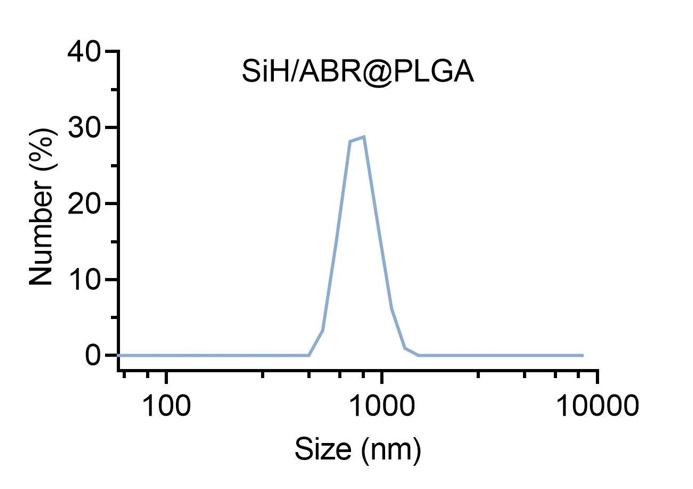


**Figure S2.** Hydrodynamic diameter of SiH/ABR@PLGA measured by DLS.


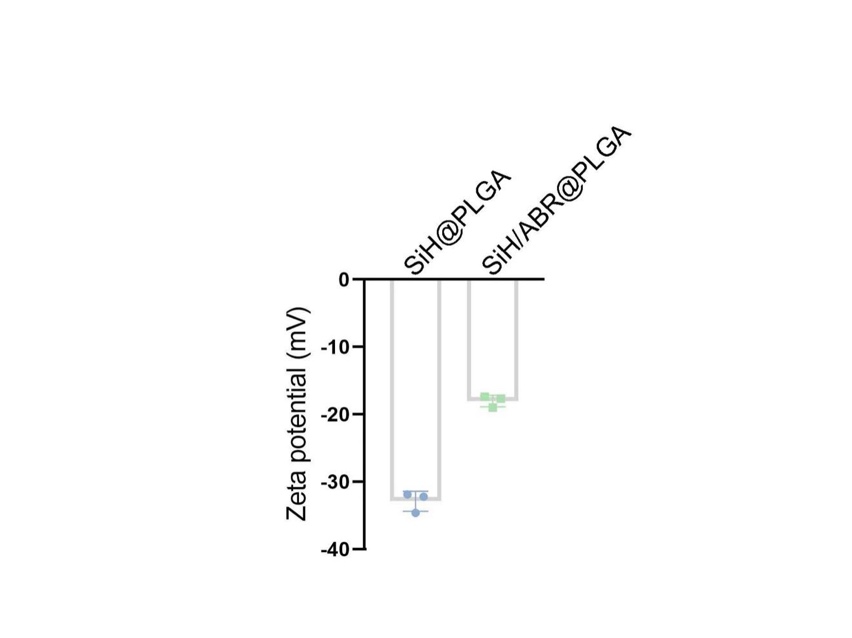


**Figure S3.** Zeta potentials of SiH@PLGA and SiH/ABR@PLGA.


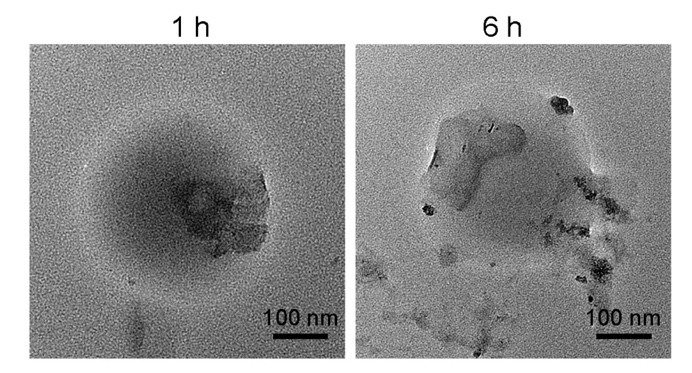


**Figure S4.** TEM images of SiH/ABR@PLGA after dispersed in PBS for 1 h or 6 h.


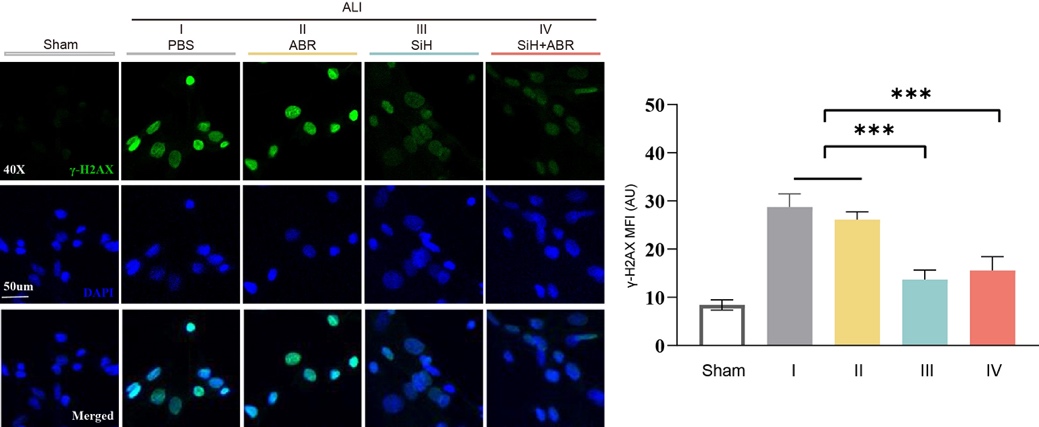


**Figure S5.** DNA impairment indicated by γ-H2AX levels of lung epithelial cells treated with ABR@PLGA, SiH@PLGA, or SiH/ABR@PLGA in the presence of H_2_O_2._ The γ-H2AX MFI indicated that the protective effects were attributed to SiH in vitro, while the ABR showed negligible role in ROS scavenging in vitro. I-ALI + PBS-treated group; II-ALI + ABR/@PLGA-treated group; III-ALI + SiH@PLGA-treated group; IV-ALI + SiH/ABR@PLGA-treated group.


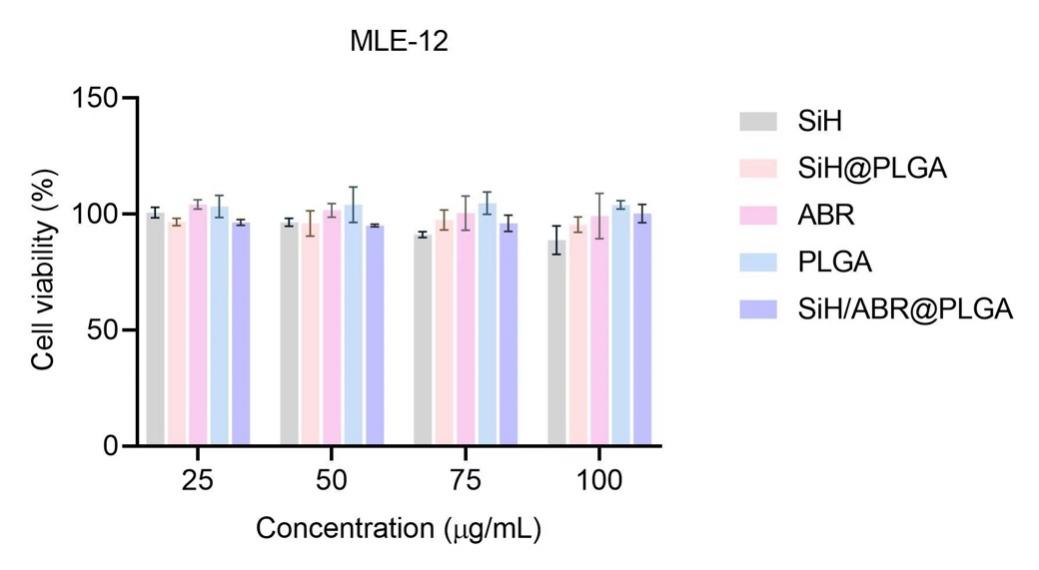


**Figure S6.** Cell viabilities of MLE-12 after being incubated with different concentrations of SiH, SiH@PLGA, ABR, PLGA, and SiH/ABR@PLGA for 24 h. The concentrations indicate the Si concentration in SiH/ABR@PLGA group, and the concentrations of other components are kept consistent with those in SiH/ABR@PLGA group.


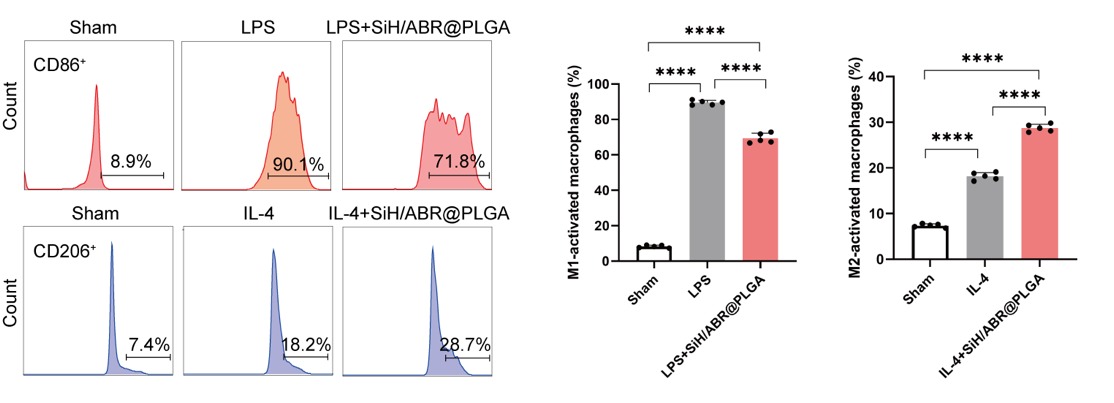


**Figure S7.** The flow cytometry analysis indicated the SiH/ABR@PLGA inhibited the M1 polarization and promoted the M2 polarization of macrophage *in vitro.*

**
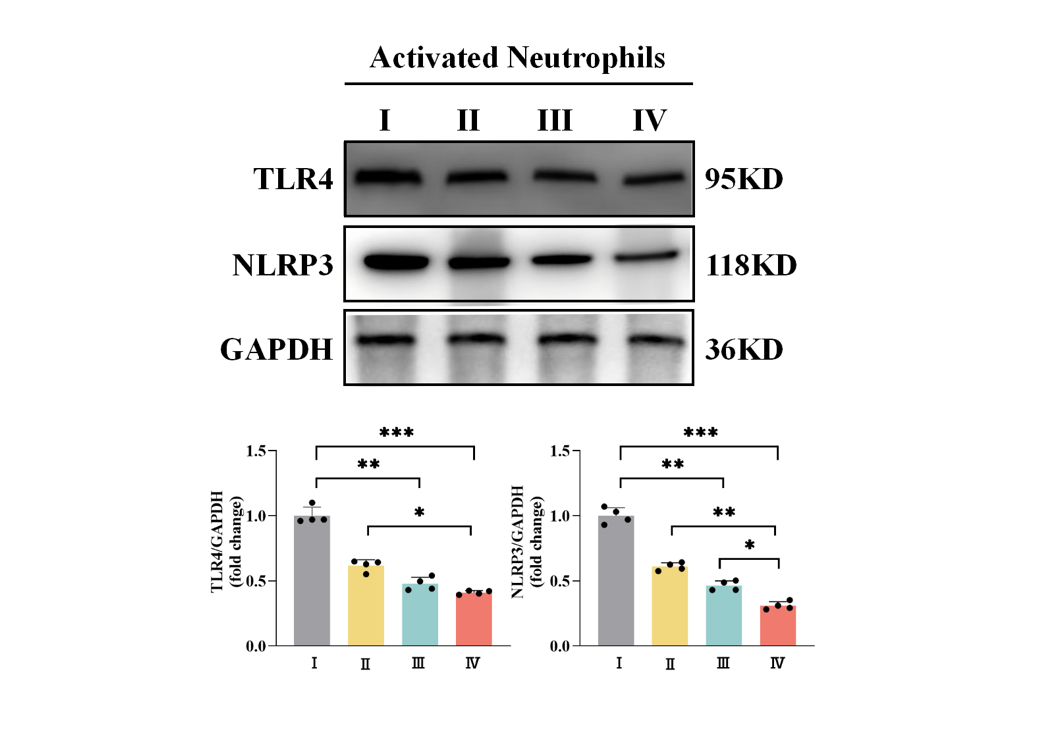
**

**Figure S8.** The TLR4 and NLRP3 inflammasome expression in activated neutrophils was inhibited under the treatments of ABR@PLGA, SiH@PLGA and SiH/ABR@PLGA.


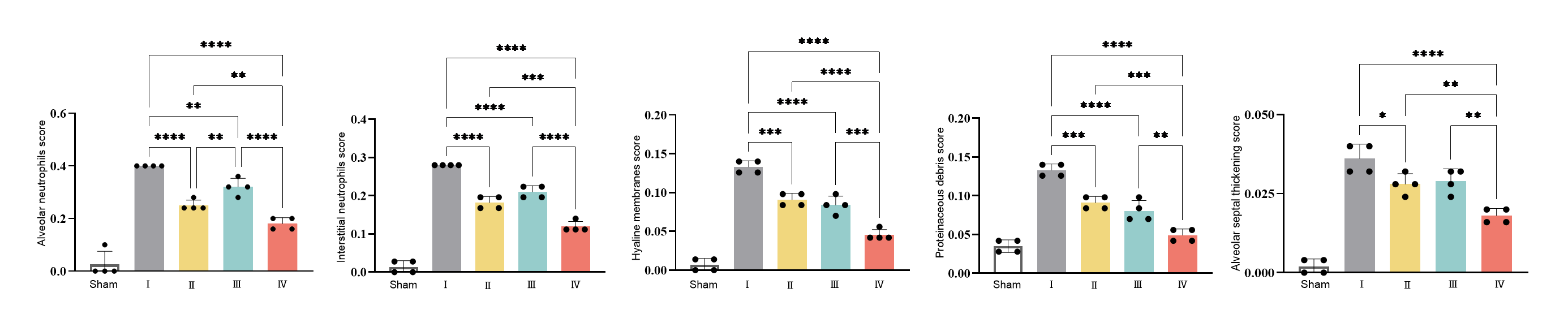


**Figure S9.** The five features of total lung injury score of ALI mice. All the nanocomplexes led to the restoration of lung injury, and the SiH/ABR@PLGA-treated group exhibited significant improvements as compared to the ABR@PLGA- and SiH@PLGA- treated groups. Data are represented as mean±SD. * indicted P <0.05, ** indicted P <0.01, *** indicted P <0.001, and **** indicted P <0.0001. I-ALI + PBS-treated group; II-ALI + ABR@PLGA-treated group; III-ALI + SiH@PLGA- treated group; IV-ALI + SiH/ABR@PLGA-treated group.


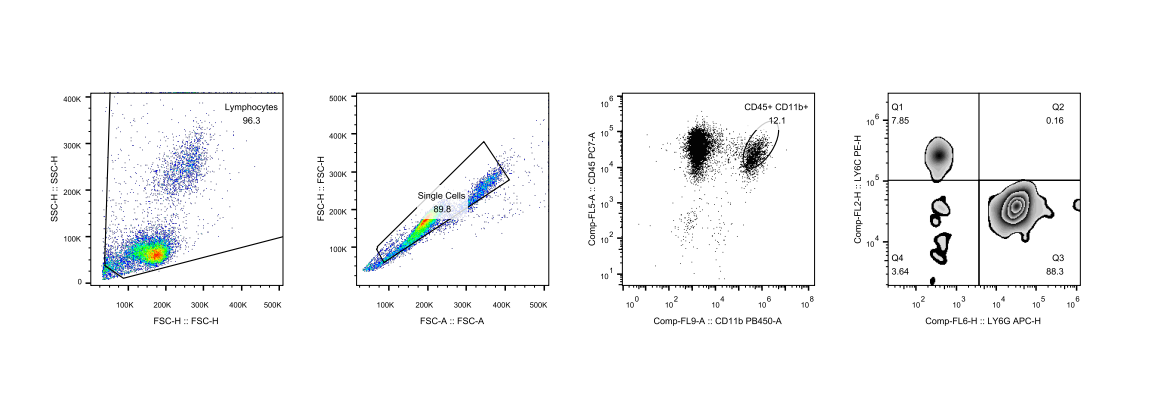


**Figure S10.** The gating strategy of the peripheral leukocytes in the blood.


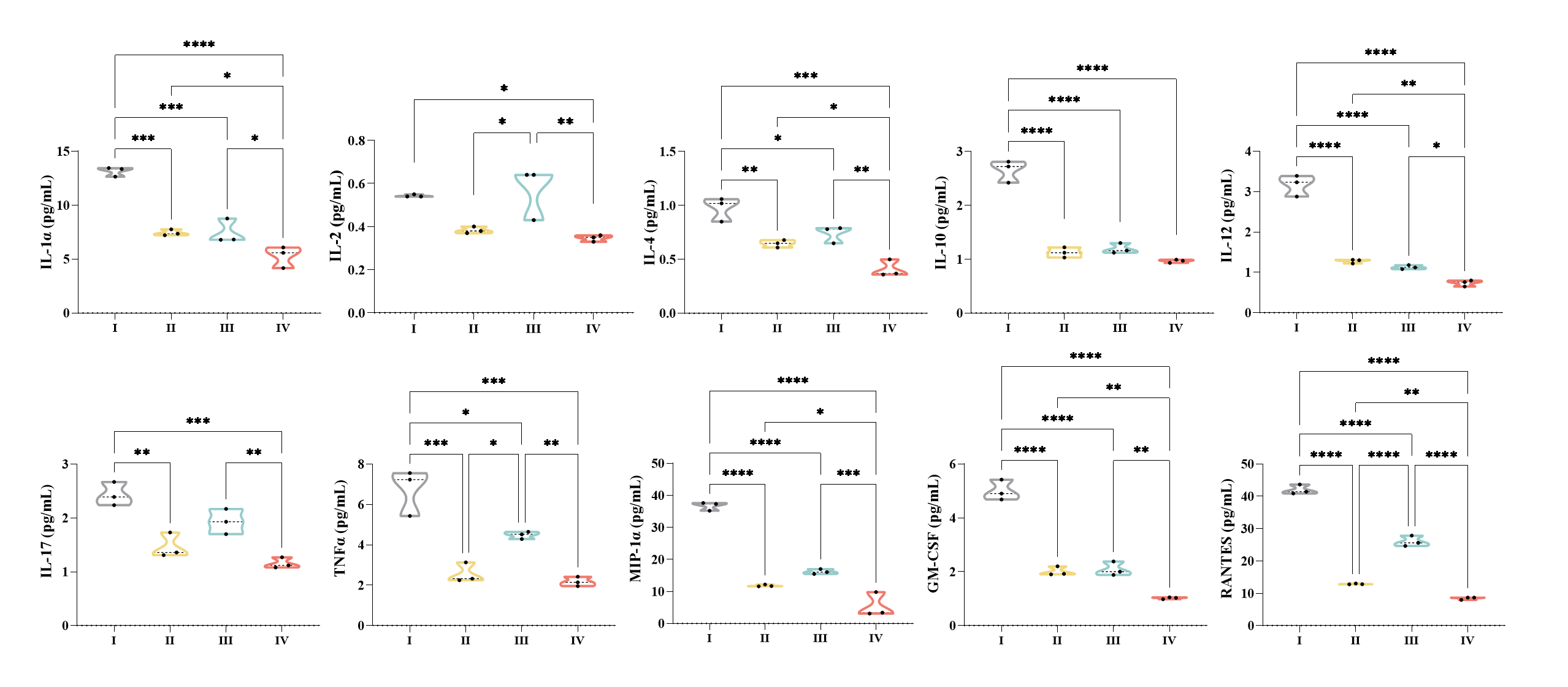


**Figure S11.** The quantification of multiple pro-inflammatory cytokines in the serum of ALI mice. All the nanocomplexes showed significant effects in inhibiting the systemic inflammation, and the SiH/ABR@PLGA displayed stronger effects when comparing to the single treatment. Data are represented as mean±SD. * indicted P <0.05, ** indicted P <0.01, *** indicted P <0.001, and **** indicted P <0.0001. I-ALI + PBS-treated group; II-ALI + ABR@PLGA-treated group; III-ALI + SiH@PLGA-treated group; IV-ALI + SiH/ABR@PLGA-treated group.


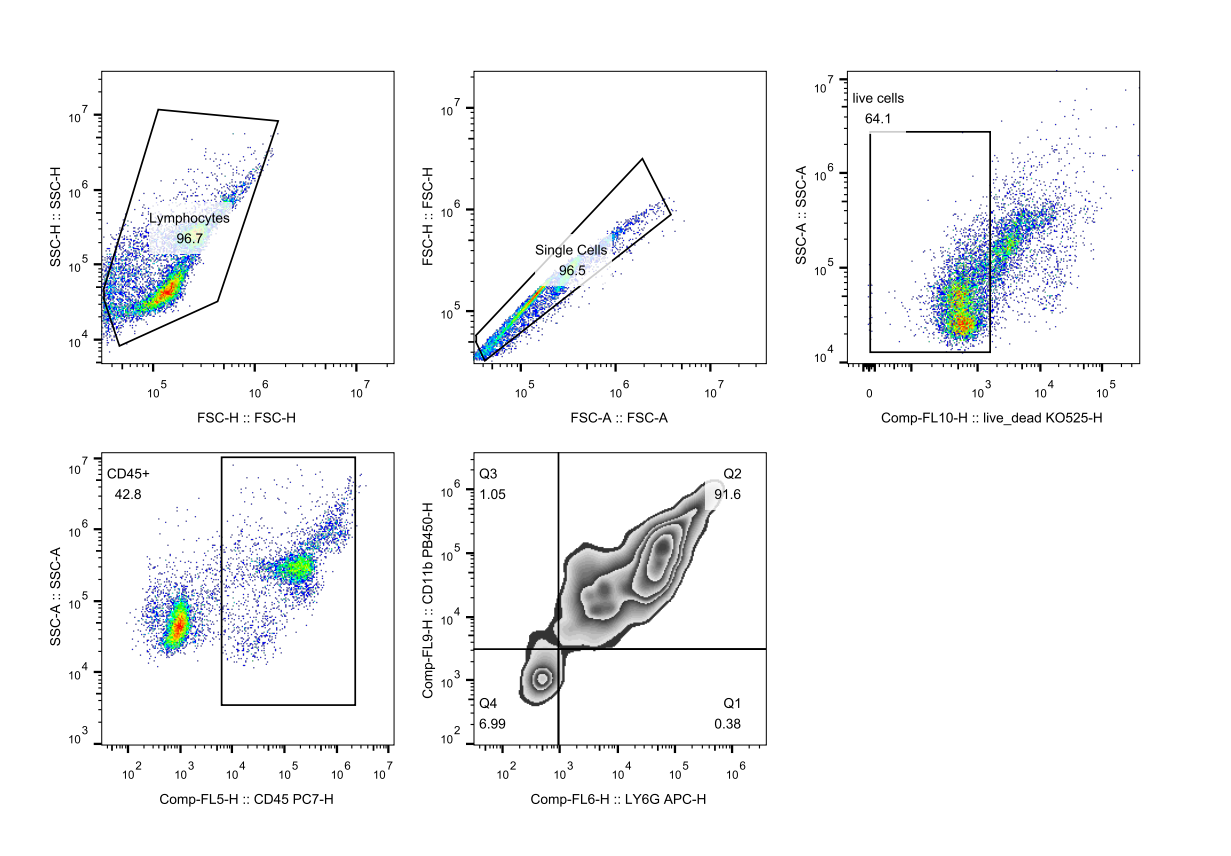


**Figure S12.** The gating strategy of the inflammatory cells in the BALF.


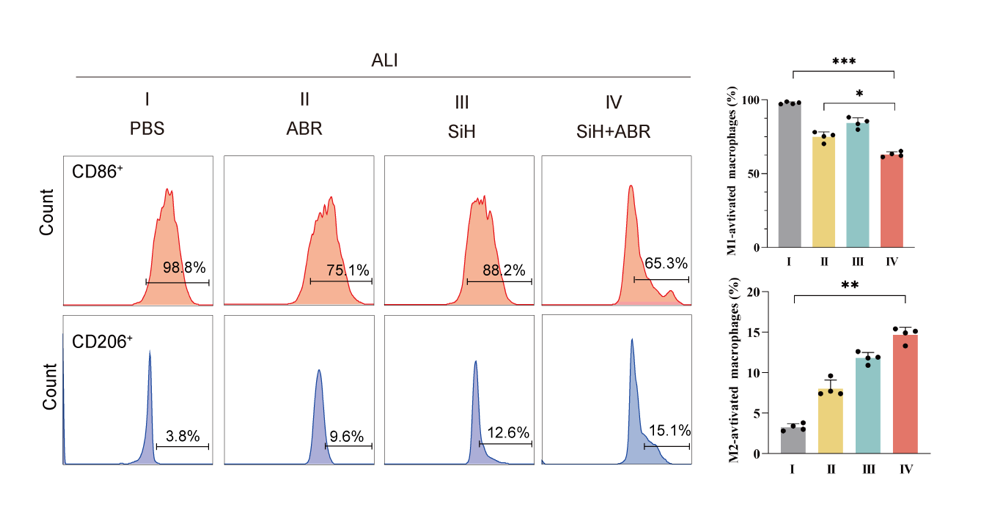


**Figure S13.** The proportion of M1 and M2 activated macrophages in the BALF of ALI mice under the treatment of ABR@PLGA, SiH@PLGA and SiH/ABR@PLGA. Data are represented as mean±SD. * indicted P <0.05, ** indicted P <0.01, and *** indicted P <0.001. I-ALI + PBS-treated group; II-ALI + ABR@PLGA-treated group; III-ALI + SiH@PLGA- treated group; IV-ALI + SiH/ABR@PLGA-treated group.


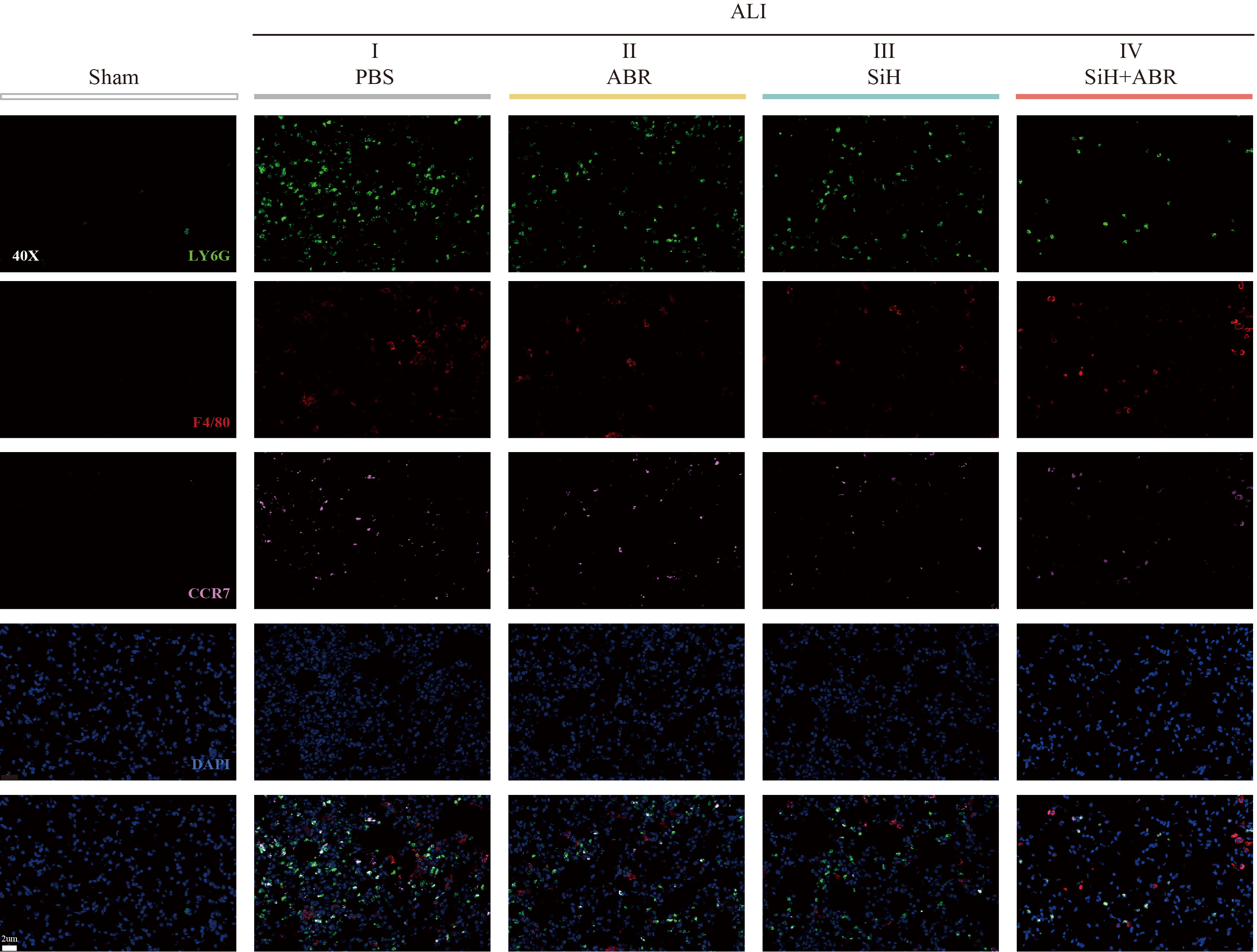


**Figure S14.** The infiltration of inflammatory cells in the lung tissue. The expressions of LY6G, F4/80 and CCR7 were significantly reduced by the treatment of all the nanocomplexes. Particularly, the SiH/ABR@PLGA displayed the the most potent inhibition effects. I- ALI + PBS- treated group; II-ALI + ABR@PLGA-treated group; III-ALI + SiH@PLGA-treated group; IV-ALI + SiH/ABR@PLGA-treated group.


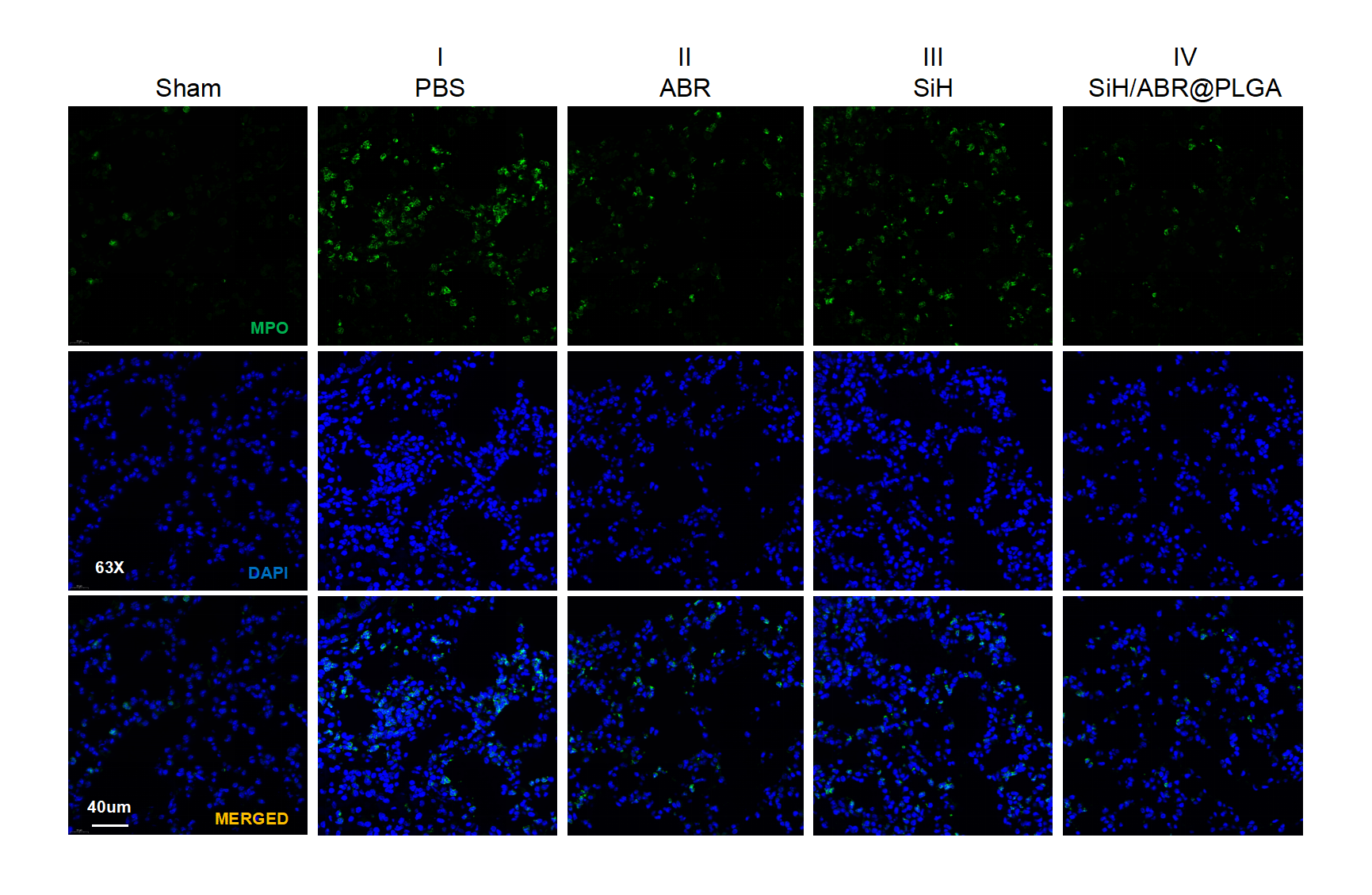


**Figure S15.** The infiltration and activation of neutrophil in the lung tissue indicated by the immunofluorescence analyses. The expressions of MPO (green) were significantly reduced by the treatment of all the nanocomplexes. Particularly, the SiH/ABR@PLGA displayed the the most potent inhibition effects. I- ALI + PBS- treated group; II- ALI + ABR@PLGA-treated group; III- ALI + SiH@PLGA-treated group; IV- ALI + SiH/ABR@PLGA-treated group.


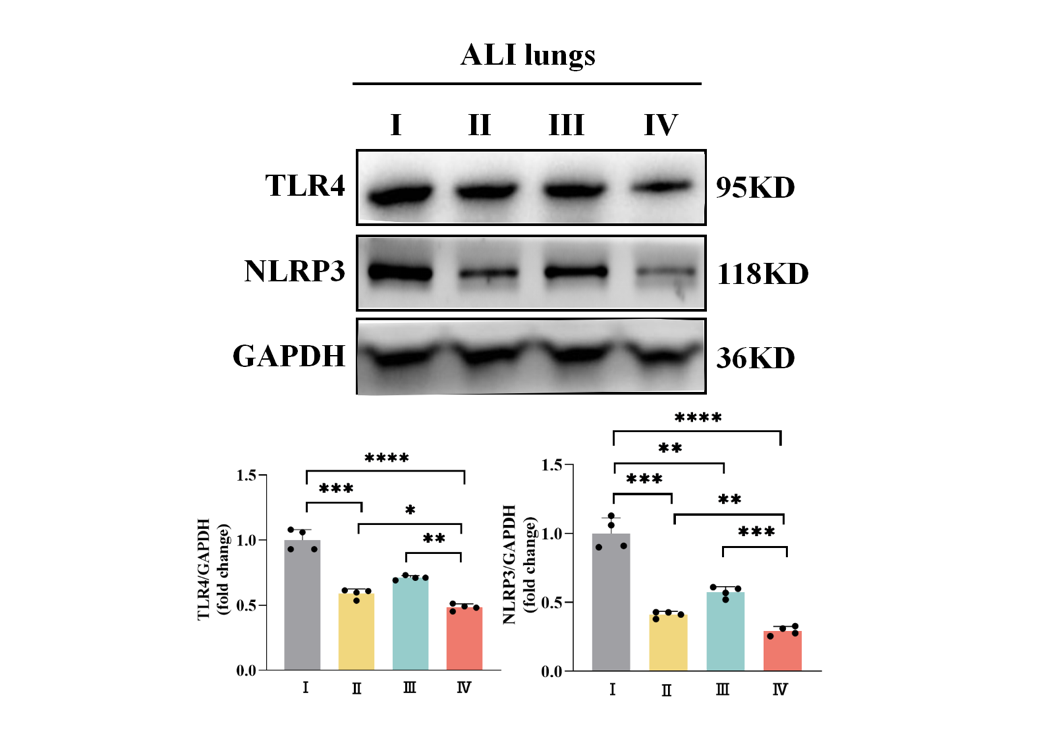


**Figure S16.** The TLR4-NLRP3 Inflammasome signaling in ALI lung was significantly inhibited upon the treatments of ABR@PLGA, SiH@PLGA and SiH/ABR@PLGA.


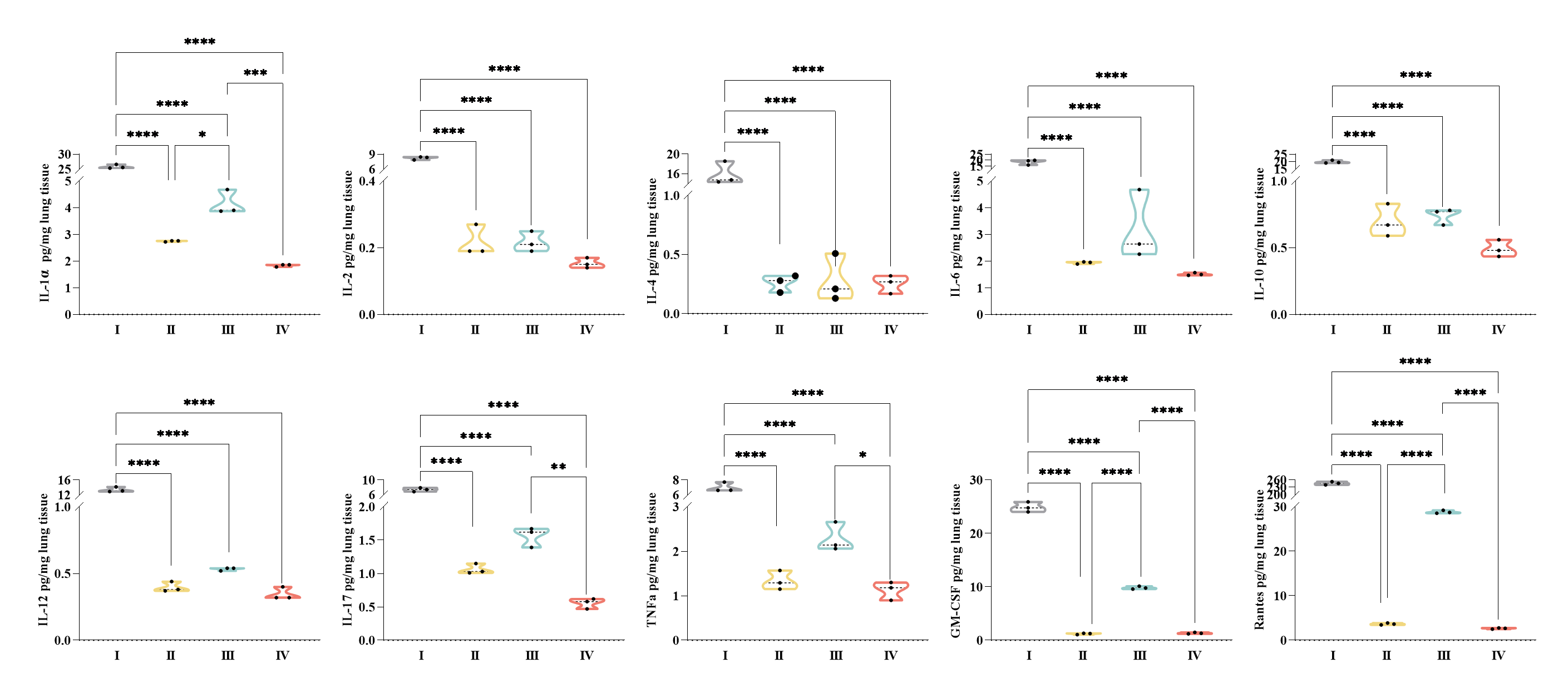


**Figure S17.** The quantification of multiple pro-inflammatory cytokines in the lung tissue of ALI mice. All the nanocomplexes showed significant effects in inhibiting the local inflammation, and the SiH/ABR@PLGA displayed stronger effects when comparing to the single treatment. Data are represented as mean ± SD. * indicted P <0.05, ** indicted P <0.01, *** indicted P <0.001, and **** indicted P <0.0001. I- ALI + PBS-treated group; II-ALI + ABR/@PLGA-treated group; III-ALI + SiH/@PLGA-treated group; IV-ALI + SiH/ABR@PLGA-treated group.


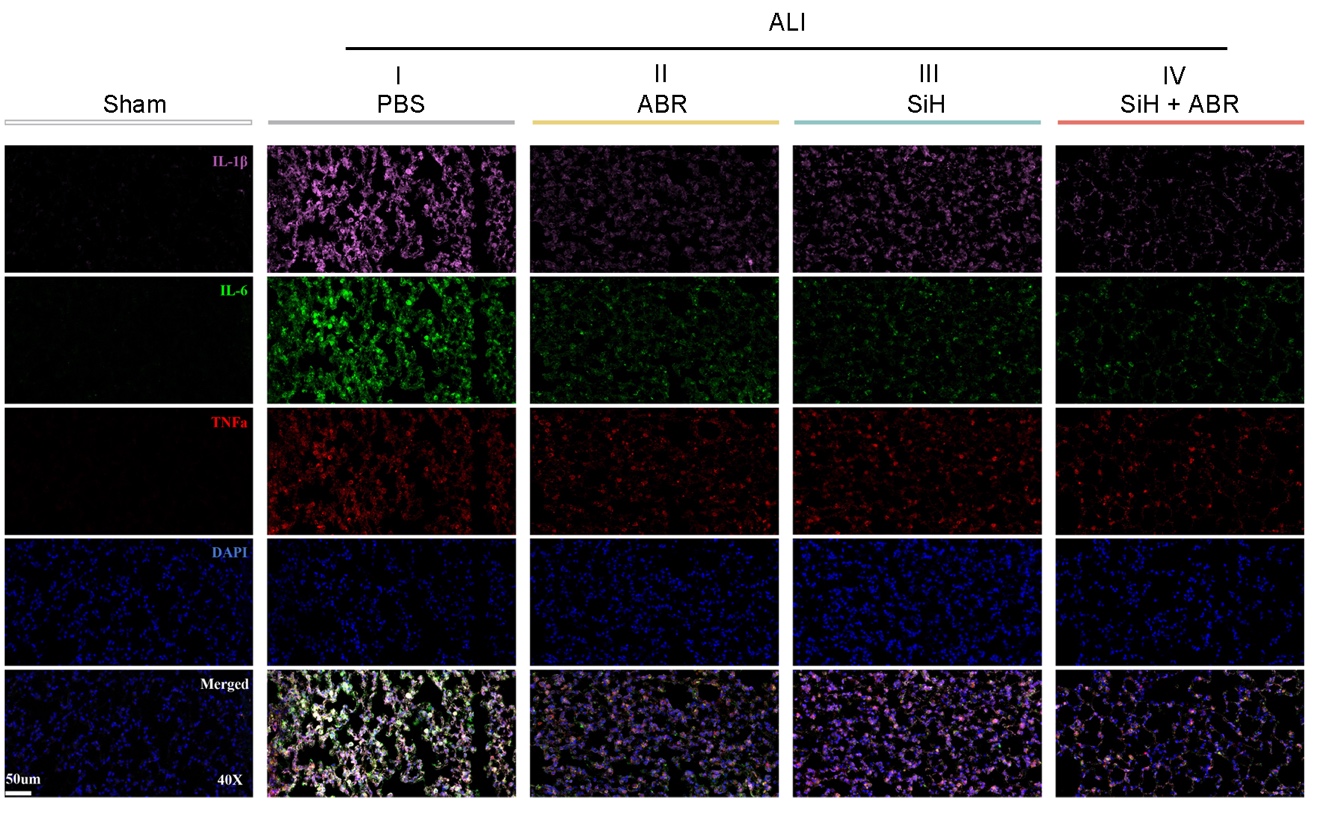


**Figure S18.** The expression of cytokines in the lung tissue. The expressions of IL-1β, IL-6 and TNFa were significantly reduced by the treatment of all the nanocomplexes. Particularly, the SiH/ABR@PLGA displayed the the most potent inhibition effects. I-ALI+PBS-treated group; II-ALI + ABR@PLGA-treated group; III-ALI + SiH/@PLGA-treated group; IV-ALI + SiH/ABR@PLGA-treated group.
